# Constrained Carbon Partitioning: A Self‐Trained Physics‐Informed Machine Learning Model Refines GPP Estimates From Eddy Covariance Measurements

**DOI:** 10.1111/gcb.70886

**Published:** 2026-05-11

**Authors:** Sadegh Ranjbar, Ankur R. Desai, Sophie Hoffman, Einara Zahn, Elie Bou‐Zeid, Paul C. Stoy

**Affiliations:** ^1^ Department of Biological Systems Engineering University of Wisconsin – Madison Madison Wisconsin USA; ^2^ Department of Atmospheric and Oceanic Sciences University of Wisconsin – Madison Madison Wisconsin USA; ^3^ Department of Civil and Environmental Engineering Princeton University Princeton New Jersey USA; ^4^ Department of Earth and Environmental Science University of Pennsylvania Philadelphia Pennsylvania USA

**Keywords:** carbon partitioning, ecosystem respiration, Eddy covariance, gross primary productivity, knowledge‐guided machine learning, Kok effect, NEON, stomatal physiology

## Abstract

Gross primary productivity (GPP) is the largest term in the global carbon budget but cannot be directly observed. We present a knowledge‐guided machine learning (KGML) framework that partitions eddy covariance‐measured net ecosystem exchange (NEE) into gross primary production (GPP) and ecosystem respiration (RECO) with partitioned water vapor fluxes and CO_2_ flux source areas from 36 U.S. National Ecological Observatory Network (NEON) towers. The KGML is guided by hard physical constraints that enforce mass balance and ‘soft’ theoretical expectations including optimal stomatal response to vapor pressure deficit (VPD) and links between GPP and transpiration (T) through stomatal function. The model achieves strong physical consistency (NEE *R* ^2^ = 0.99) while capturing expected ecophysiological relationships including GPP‐T coupling (*R* ^2^ = 0.58) and stomatal responses to light and VPD. Compared to conventional partitioning methods, KGML infers lower GPP and RECO estimates on average, with the largest negative biases occurring at low light levels (0–200 μmol photons m^−2^ s^−1^). These differences likely reflect a combination of mechanisms including light‐induced respiration suppression consistent with the Kok effect, stomatal‐transpiration coupling constraints, and dynamic allocation between respiration components. The flux differences vary across plant functional types (PFTs), where forested ecosystems (deciduous broadleaf, evergreen needleleaf, and mixed), savannas and grasslands show the largest negative annual GPP deviations (−10% to −18% versus nighttime partitioning), while croplands and open shrublands show moderate negative deviations (−5% to −10%). The lower GPP estimates by PFT are closer to those inferred by Keenan et al. that explicitly considers limitations on RECO from the Kok effect. We discuss implications for our understanding of ecosystem and global carbon cycle processes, as well as ways to further benefit from the full information content of eddy covariance observations by combining physics with knowledge of biological processes.

## Graphical abstract

Plant canopy carbon dioxide uptake by photosynthesis, called gross primary productivity (GPP), is the largest term in the global carbon cycle but cannot be directly measured at the ecosystem scale. We use a unique dataset of ecosystem net carbon dioxide exchange with a knowledge‐guided machine learning model that treats GPP as a ‘hidden’ process and uses physical laws and biological rules to discover it. We find that GPP follows daily patterns that are consistent with biological mechanisms that it was not trained to learn and is slightly lower than other estimates, which revises our understanding of carbon cycling.

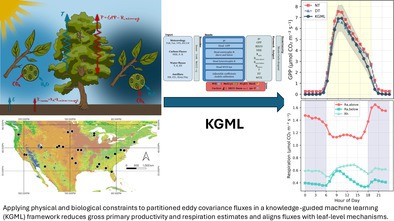

## 1 Introduction

It is critical to quantify ecosystem carbon inputs via gross primary productivity (GPP) to understand the role that ecosystems play in the climate system and opportunities to enhance natural climate solutions (Novick 2022; Novick et al. 2022). Eddy covariance has quickly become the standard methodology for measuring the net ecosystem‐atmosphere flux of carbon dioxide (NEE) at the ecosystem scale (D. Baldocchi 2008, 2014; D. D. Baldocchi 2020), but this net flux must be “partitioned” into GPP and ecosystem respiration (RECO) to estimate the biological processes that comprise it (Reichstein et al. 2012). There are multiple established approaches for doing so, all of which provide different estimates of the magnitude of GPP and its sensitivity to environmental drivers (Desai et al. 2008), and thereby different interpretations of the role of terrestrial ecosystems in the carbon cycle.

The most widely used partitioning methods rely on the fact that during nighttime, GPP is zero in ecosystems without CAM vegetation such that measured NEE is composed entirely of RECO (Reichstein et al. 2005). These nighttime measurements are then used to establish a model for RECO that is a function of temperature, and sometimes other variables like soil moisture or recently assimilated carbon (Migliavacca et al. 2011; Noormets et al. 2008). The RECO model is subsequently applied to estimate GPP during daytime as the difference between measured NEE and the RECO model by definition (NEE = RECO—GPP). This “nighttime” approach assumes that the processes controlling RECO are the same during day and night and relies on nighttime eddy covariance measurements that only include periods with sufficient turbulent intensity, as often approximated using a minimum friction velocity (u*) threshold (Papale et al. 2006) to exclude strongly stable conditions that pose challenges to the eddy covariance method.

‘Daytime’ partitioning approaches use the intercept of light response curves to estimate RECO and subsequently GPP (Lasslop et al. 2010), and thereby seek to minimize the nighttime measurement problem, but rely on an intercept parameter to infer RECO that may be difficult to estimate accurately using common light response curves (Gilmanov et al. 2003; Stoy et al. 2006). Additional partitioning approaches have been developed that adjust for missing measurements due to the strong suppression of turbulence that often occurs during early evening at a time when soil temperatures tend to be relatively warm which favors RECO (Van Gorsel et al. 2007) or account for excluded processes like the suppression of autotrophic respiration by light (Heskel et al. 2013; Keenan et al. 2019; Wehr et al. 2016) known as the Kok effect (Kok 1949; Yin et al. 2020). The Kok effect is typically observed at very low irradiance near the light‐compensation point, generally within approximately 0–20 μmol m^−2^ s^−1^ PAR, where light‐induced suppression of respiration causes a change in the slope of photosynthetic light‐response curves (Heskel et al. 2013; Sharp et al. 1984; Yin et al. 2020). This region is often followed by transition zones extending roughly from 20 to 50 and 50 to 160 μmol m^−2^ s^−1^ PAR, where the influence of respiration suppression diminishes and the light‐limited photosynthetic response begins to dominate (Fu et al. 2012; Gauthier et al. 2018; Heskel et al. 2013; Léger‐Daigle et al. 2022). All partitioning methods rely on models for how ecosystems respond to light and temperature, which may give rise to spurious correlations as GPP and RECO respond to both (Austin and Vivanco 2006; Baldocchi et al. 2015; Tramontana et al. 2020). Partitioning models also tend to be difficult to parameterize as ecosystems change throughout the seasonal cycle and frequently give different results (Desai et al. 2008; Stoy et al. 2006), making it unclear how to best combine eddy covariance observations and models to estimate GPP.

Machine learning (ML) approaches for flux partitioning have evolved from pure data‐driven methods to increasingly sophisticated hybrid frameworks that combine physical constraints with ML flexibility. Early ML partitioning efforts focused on learning empirical relationships from eddy covariance data (Tramontana et al. 2020), while more recent hybrid physics‐ML approaches have sought to address fundamental partitioning challenges through various strategies. Deep state space models have been used to learn dynamical models of RECO from nighttime measurements while accounting for temporal dependencies and uncertainty (Trifunov et al. 2021). Causal inference frameworks combined with double machine learning have been applied to flux partitioning to mitigate equifinality, where multiple parameter sets describe data equally well, and avoid biases introduced by regularization techniques (Cohrs et al. 2024). Despite these advances, hybrid modeling approaches face persistent challenges including the appropriate selection of input variables, which can lead to models that are “right for the wrong reasons” and compromise interpretability (Cohrs et al. 2024), and the reliance on prescribed functional forms for light and temperature responses that may not capture ecosystem‐specific behaviors.

Beyond CO_2_ flux partitioning, hybrid ML frameworks have also been applied more broadly to better represent ecosystem CO_2_ dynamics, including spatializing continental‐scale fluxes from flux tower data (Papale and Valentini 2003), evaluating the role of spatial sampling in artificial neural network based upscaling of GPP and latent heat fluxes (Papale et al. 2015), replacing rigid parameterizations of GPP and transpiration modules within Earth system models using neural networks trained on eddy covariance data (ElGhawi et al. 2025), quantifying drought legacy effects on GPP across global biomes using long‐term flux tower records (Yu et al. 2025), and generating global upscaled GPP products that incorporate the CO_2_ fertilization effect previously omitted from data‐driven estimates (Kang et al. 2025). A recent mini‐review by Jin et al. (2026) synthesizes emerging hybrid and knowledge‐guided ML strategies for ecosystem modeling, highlighting advances in physics‐informed learning, causal embedding, and theory‐constrained architectures, and calls for frameworks that move beyond post hoc corrections toward models that integrate mechanistic principles directly within training to improve interpretability, extrapolation, and ecological realism.

A particularly challenging issue across partitioning methods, both traditional model‐based and hybrid ML approaches, is their treatment of the Kok effect, whereby autotrophic respiration is suppressed by light during daytime (Heskel et al. 2013; Keenan et al. 2019; Kok 1949; Tramontana et al. 2020; Wehr et al. 2016; Yin et al. 2020). Most partitioning approaches either ignore this process or attempt to correct for it through post hoc adjustments, potentially leading to systematic biases in both GPP and RECO estimates and misrepresentation of their environmental sensitivities. We argue that ML has untapped promise for discovering such biological processes by treating GPP and its interactions with respiration as ‘latent’ (invisible) processes within eddy covariance measurements of NEE. Our knowledge‐guided machine learning (KGML) approach is designed to advance beyond existing hybrid methods in several key ways. First, rather than prescribing functional forms for processes like light response or temperature sensitivity a priori, our framework allows the model to learn nonlinear responses, including potential suppression of respiration components during daytime, directly from observations while being constrained by ‘hard’ physical rules for carbon dioxide mass balance conservation in the model architecture and ‘soft’ biological guidance from established theory embodied in loss functions. Second, by explicitly treating GPP as a latent variable that must be inferred jointly with RECO within the mass balance constraint, our approach accounts for coupled responses to environmental drivers without requiring separate parameterizations for each flux component. We apply this approach to infer GPP from eddy covariance measurements across the NEON network with source areas partitioned using high frequency eddy covariance measurements (Zahn and Bou‐Zeid 2024a) to allow the model to reveal ecosystem‐specific carbon cycle dynamics without explicit parameterization. A motivation for our approach with hard physical and soft biological constraints is a quote popularized by Dennis Baldocchi: Physics wins, biology is how it's done.

## 2 Materials and Methods

### 2.1 Theoretical Overview

**FIGURE 1:**
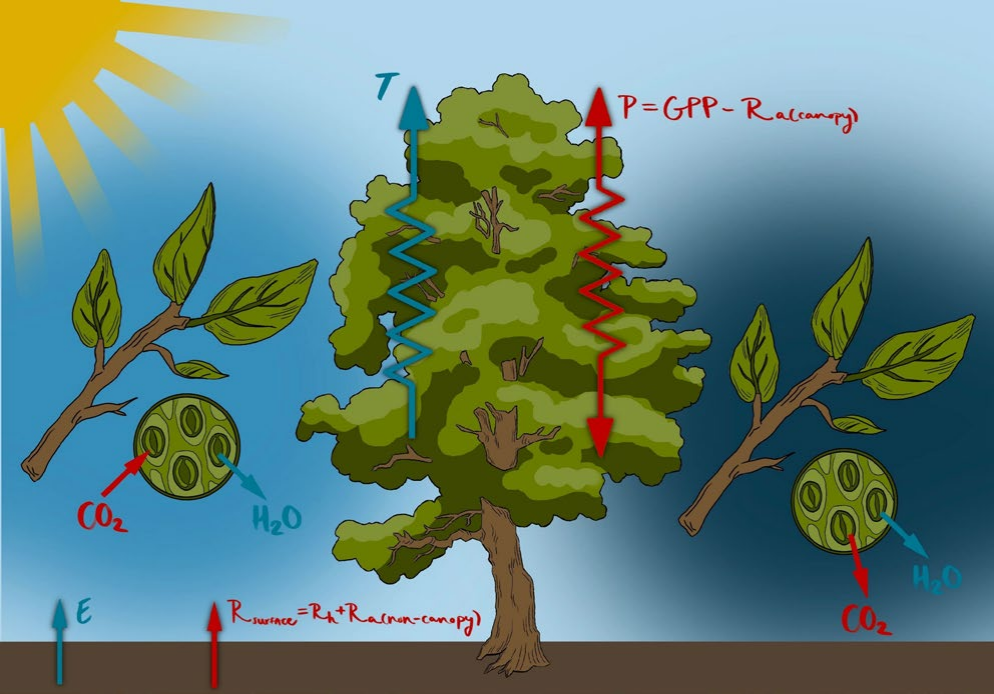
The coupled CO_2_ and H_2_O fluxes of an ecosystem, with the terms that can be inferred from high frequency flux partitioning methods. These include evapotranspiration (E) and transpiration (T) that sum to measured evapotranspiration (ET) and net primary production (P), itself equal to GPP minus autotrophic respiration from the canopy (R_a(canopy)_), plus surface respiration (R_surface_) equal to heterotrophic respiration (R_h_) and autotrophic respiration from non‐canopy components (R_a(non‐canopy)_) that altogether sum to measured net ecosystem exchange (NEE). The left panel (daytime) depicts conditions under sunlight, where photosynthesis dominates, leading to CO_2_ uptake and substantial H_2_O loss via transpiration through open stomata (Medlyn et al. 2011; Wang et al. 2021). The right panel (nighttime) shows CO_2_ release driven by respiration and minimal H_2_O loss, as the stomata are partially closed to conserve water (Caird et al. 2007).

Our KGML framework is designed to integrate process‐based understanding of ecosystem carbon and water fluxes that can be measured using eddy covariance and inferred using high frequency partitioning of flux sources (Figure 1) with a data‐driven modeling approach that can flexibly capture nonlinear and site‐specific relationships. Rather than relying solely on empirical correlations, our KGML leverages prior knowledge about physiological and biophysical processes, such as carbon‐water coupling through stomata, to guide the learning of complex nonlinear relationships from observational data. This approach is designed to maintain physical plausibility while capturing site‐specific variability across diverse ecosystems.

To ensure physically consistent predictions, the KGML framework incorporates hard and soft constraints that guide the learning process as described in more detail in Section 2.3. Hard constraints enforce fundamental physical and biological rules, such as non‐negativity of stomatal conductance, GPP, and RECO, as well as carbon dioxide mass balances. These ensure that the model outputs remain physically plausible under all conditions. Soft constraints encourage the model to follow known ecological patterns without strictly enforcing them. Examples include expected relationships between GPP and transpiration through stomatal function, water‐use efficiency responses to key drivers including optimal stomatal behavior under varying vapor pressure deficit (Medlyn et al. 2011), and nighttime suppression of photosynthesis (De Kauwe et al. 2019; Krich et al. 2022).

### 2.2 Data

We used eddy covariance (EC) observations from the National Ecological Observatory Network (NEON), a continental‐scale, long‐term, open‐access environmental monitoring network across the United States (Keller et al. 2008). For this study, we selected 35 sites that provided high frequency (20 Hz) eddy covariance data necessary for high frequency flux partitioning, spanning diverse ecosystems, including evergreen and deciduous forests, grasslands, shrublands and crops, for which high‐quality GPP and RECO estimates from conventional partitioning methods (described) could be calculated. Each tower provides continuous, half‐hourly measurements of net ecosystem exchange (NEE), LE and sensible heat flux (H), incident and outgoing shortwave and longwave radiation, soil water content (SM), air temperature (TA), relative humidity (RH), and other ancillary meteorological variables. All flux data were processed using standardized NEON protocols, including friction velocity (u*) threshold filtering to remove unreliable nighttime measurements (Pastorello et al. 2020; Sturtevant et al. 2022). To compare our KGML‐based estimates, we used the Python library *hesseflux* (Cuntz 2020) to derive conventional nighttime‐partitioned GPP and RECO (GPP_NT_ and RECO_NT_, Reichstein et al. (2005)) and daytime‐partitioned GPP and RECO (GPP_DT_ and RECO_DT_, Lasslop et al. (2010)).

**FIGURE 2:**
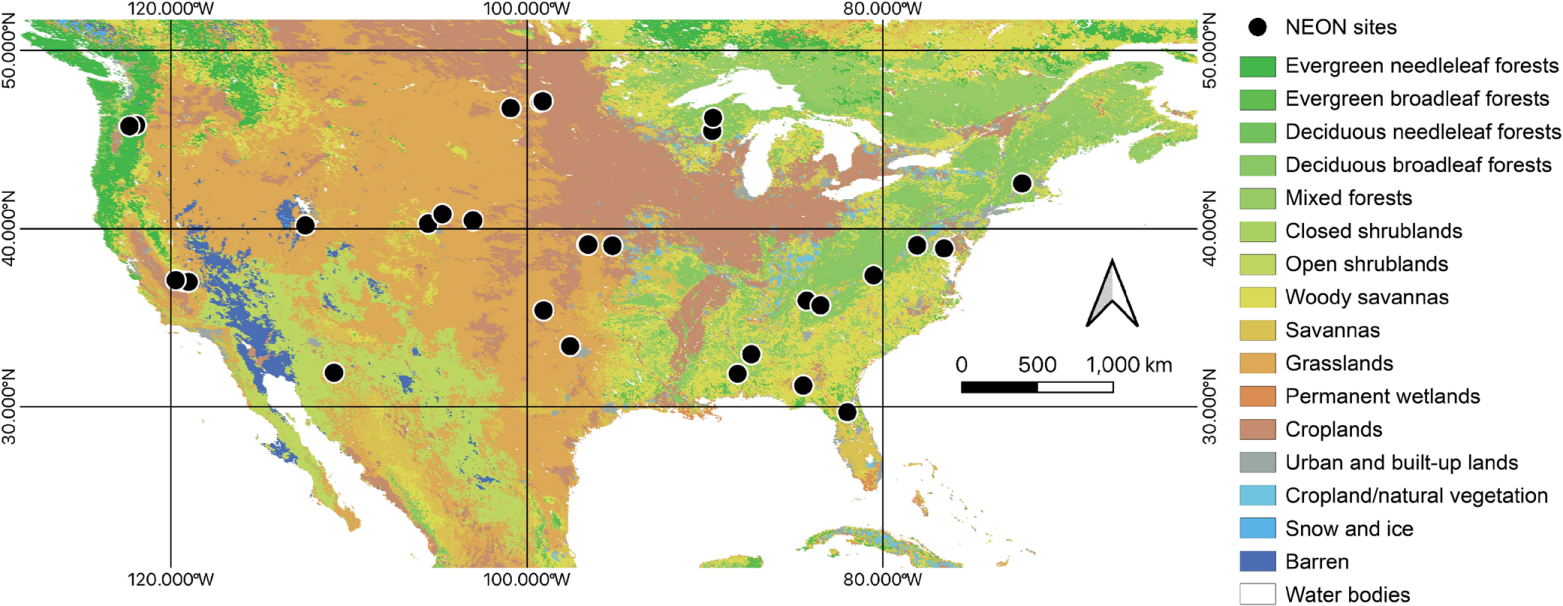
The spatial distribution of the NEON sites included in this study, overlaid on IGBP vegetation categories.

We used high frequency partitioned carbon dioxide and water fluxes in our KGML modeling. The canopy versus surface components of CO_2_ and water fluxes (Figure 1) were obtained using conditional eddy accumulation (cea, Zahn et al. 2022) applied across the NEON network (Figure 2). The cea approach separates eddy covariance‐measured evapotranspiration into canopy transpiration (T) and soil/surface evaporation (E) and NEE into net canopy (P_cea_) and surface (R_cea_) components using minimal assumptions about canopy physiology (e.g., it does not require water use efficiency estimates) and turbulent exchange (i.e., it does not rely on Monin‐Obukhov Similarity Theory). We compare results against flux variance similarity (fvs, Scanlon and Kustas 2010, 2012; Scanlon and Sahu 2008) with water use efficiency estimated using optimality theory (Scanlon et al. 2019). While fvs benefits from its explicit water‐use efficiency constraints, uncertainties in water use efficiency (WUE) for C_4_ vegetation limit its universal applicability and the solution rarely converges if atmospheric stability is not near neutral such that it provides fewer estimates. Therefore, we primarily present results based on cea and include fvs results in Appendix S1 for completeness. The combined dataset spans 2019–2023 and provides synchronized half‐hourly observations of net carbon and water fluxes and their net source and sink areas within the ecosystem (Zahn and Bou‐Zeid 2024b, Figure 2), as well as relevant meteorological drivers for model input. Preprocessing steps, including outlier removal, filtering, and temporal alignment, were applied consistently across sites (Chu et al. 2023; Pastorello et al. 2020). Only variables relevant for NEE flux partitioning and the KGML model, as listed in Table 1, were retained. Preprocessed data served as inputs for both the KGML model and conventional flux partitioning baselines.

**TABLE 1:** Predictors used in KGML modeling. Soil moisture (SM) at the second level below the surface was found to be more temporally consistent across the study sites and is used here.

Category	Variable	Description
Carbon fluxes	NEE	Net ecosystem exchange (μmol CO_2_ m^−2^ s^−1^, observed)
	P_cea_, P_fvs_	Net stomatal CO_2_ flux (μmol CO_2_ m^−2^ s^−1^, partitioned)
	R_cea_, R_fvs_	Net non‐stomatal CO_2_ flux (μmol CO_2_ m^−2^ s^−1^, partitioned)
Water fluxes	T_cea_, T_fvs_	Canopy transpiration (mmol H_2_O m^−2^ s^−1^, partitioned)
	E_cea_, E_fvs_	Soil/surface evaporation (mmol H_2_O m^−2^ s^−1^, partitioned)
	ET	Total evapotranspiration (mmol H_2_O m^−2^ s^−1^, observed)
Meteorology	PPFD	Photosynthetic Photon Flux Density (μmol m^−2^ s^−1^)
	TA	Air temperature (°C)
	VPD	Vapor pressure deficit (kPa)
	SM	Volumetric soil water content (m^3^ m^−3^) at the second level below the surface
	LE	Latent heat flux (W m^−2^)
	H	Sensible heat flux (W m^−2^)
Additional	CO_2_	Atmospheric CO_2_ mixing ratio (ppm)
	RH	Relative humidity (%)
	WS	Wind speed (m s^−1^)
	Tsonic	Sonic anemometer air temperature (°C)
	SW_IN, SW_OUT	Incoming and outgoing shortwave radiation (W m^−2^)
	LW_IN, LW_OUT	Incoming and outgoing longwave radiation (W m^−2^)
	NEE_mean	The mean NEE across the observation night and the two adjacent nights.

### 2.3 KGML Model Overview

**FIGURE 3:**
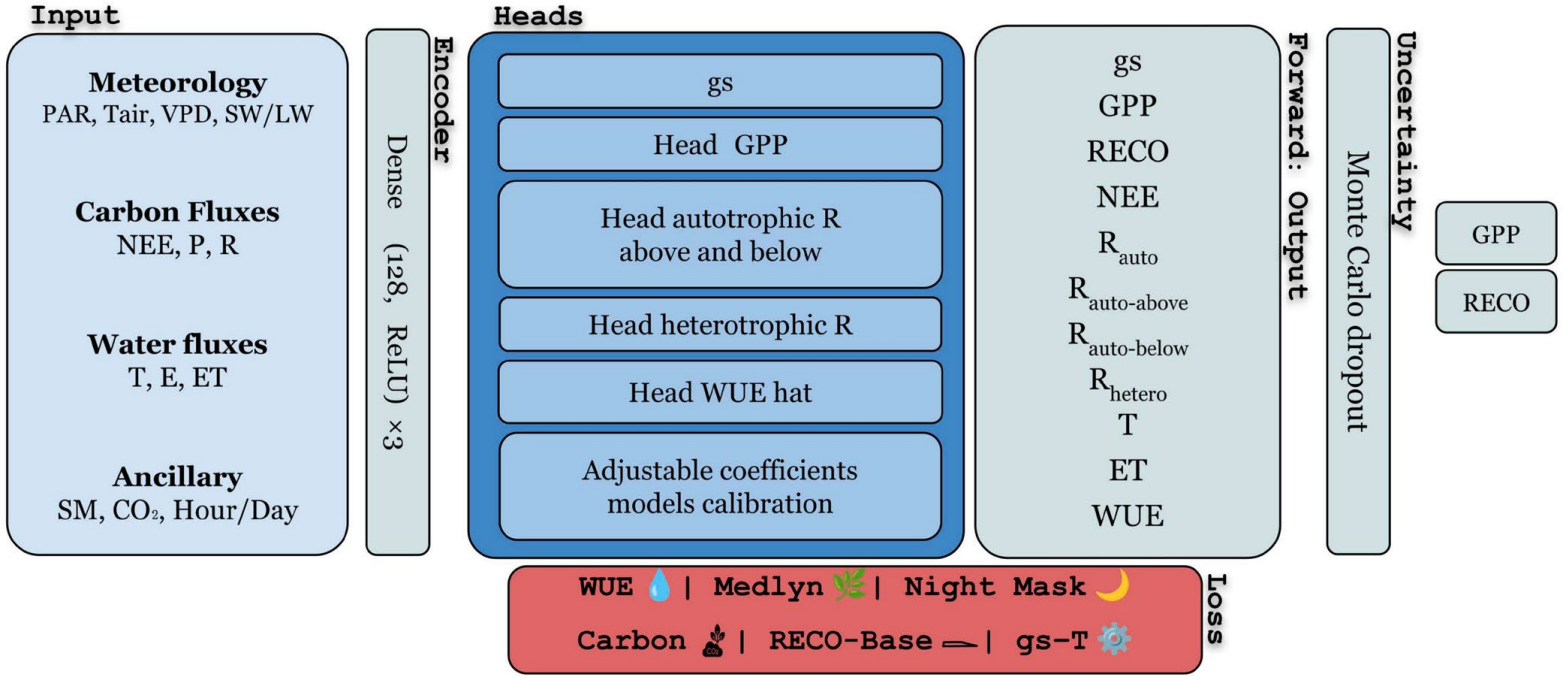
Schematic overview of the KGML modeling framework. The model receives meteorological, carbon, water, and ancillary site‐level variables as input, which are passed through a shared encoder composed of three stacked dense layers to produce a common latent representation. From this shared representation, six task‐specific heads, each being an independent sub‐network branch with its own learnable parameters, specialize in predicting a distinct biophysical process: Stomatal conductance, GPP, above‐ and below‐ground autotrophic respiration, heterotrophic respiration, WUE, and adjustable calibration coefficients. This multi‐head design allows the model to jointly learn interdependent carbon and water cycle variables while keeping their predictions partially decoupled. The forward output layer aggregates all head predictions into a full suite of carbon and water flux variables. Uncertainty in GPP and RECO is estimated through Monte Carlo dropout applied at inference time. The network is trained using a composite loss function combining physics‐based constraints to ensure predictions remain consistent with ecophysiological theory.

We developed a self‐supervised knowledge‐guided machine learning (KGML) framework to partition NEE into GPP and RECO using NEON tower data. The high‐level architecture (Figure 3) consists of:

- Shared Encoder: A multi‐layer neural encoder that transforms meteorological, flux, and ancillary data into a latent feature space to capture nonlinear interactions across environmental drivers.
- Multi‐Head Output Layer: A set of specialized heads that maps the latent representation into process‐informed outputs:
  - Stomatal conductance (gs), constrained to respond to key drivers (PAR, TA, SM, VPD).
  - GPP estimation, conditioned on gs and environmental features known to be important to ecosystem CO_2_ uptake.
  - Respiration partitioning into autotrophic aboveground (R_a,above_), autotrophic belowground (R_a,below_), and heterotrophic (R_h_) components (Figure 1).
  - A WUE prior, linking T to GPP in a physiologically realistic, non‐negative manner.
  - Adjustable coefficients, which calibrate proportionality relationships such as gs–T coupling and are consistent with optimality theory (Medlyn et al. 2011).
- Physics‐ and Ecology‐Informed Losses: The training step incorporates multiple biophysical constraints, described in Section 2.5.
- Uncertainty Estimation: Monte Carlo dropout (Gal and Ghahramani 2016) during inference provides calibrated predictive uncertainty for GPP and RECO.

Rather than relying on a single standalone process‐based model with its uncertainties, our framework integrates multiple complementary physical and eco‐physiological relationships (e.g., stomatal regulation, carbon mass balance, respiration temperature sensitivity, and water–carbon coupling) within the learning architecture. These relationships are used to define intermediate eco‐physiological variables which are explicitly predicted by dedicated model heads and propagated through physically motivated forward equations to derive carbon and water fluxes. The loss function enforces consistency on these physically derived quantities, creating a closed‐loop coupling between learned process states and physical constraints.

### 2.4 Physical Constraints and Loss Design

Hard constraints are implemented directly within the model architecture and are always enforced. For example, GPP and RECO are constrained to be non‐negative via the Softplus activation function, and predicted net ecosystem exchange (NEE) is computed as follows:

(1) $${\mathrm{NEE}}_{\text{Pred}}={\text{KGML}}_{\text{RECO}}–{\text{KGML}}_{\mathrm{GPP}}.$$

Soft constraints imposed through loss functions (L) are trained to minimize deviations from expected ecological relationships. A soft constraint involves WUE (L_WUE_), implemented by comparing the ratio of predicted GPP to transpiration with a learned WUE prior, $\hat{\mathrm{WUE}}$:

(2) $$L_{\mathrm{WUE}}=\frac{1}{N}\sum_{i}^{N}\frac{{\mathrm{GPP}}_{i}}{T_{\mathrm{cea},\;i}}-\hat{\mathrm{WUE}}$$

This encourages the model to maintain a physiologically meaningful coupling between carbon uptake and water loss while allowing local deviations (Hatfield and Dold 2019) while incorporating pre‐trained information that is consistent with KGML approaches.

Stomatal regulation is guided through a Medlyn‐based optimality loss (Medlyn et al. 2011) that aligns predicted GPP with stomatal responses to PAR, VPD, TA, and gs derived from optimality theory:

(3) $$L_{\mathrm{Med}}=\frac{1}{N}\sum_{i}^{N}\left|{\mathrm{GPP}}_{i}-k_{\mathrm{Med}}{\mathrm{gs}}_{i}\frac{{\mathrm{PAR}}_{i}}{\sqrt{{\mathrm{VPD}}_{i}}}\left(1+\beta_{T}T_{\mathrm{air},i}\right)\right|$$

where $k_{\mathrm{Med}}$ is a learnable proportionality and $\beta_{T}$ is a temperature scaling factor.

The gs‐T loss term ensures that gs and atmospheric demand for water via VPD are linked to T_cea_ (Figure 1):

(4) $$L_{\mathrm{gs}-T}=\frac{1}{N}\sum_{i}^{N}\left|k_{T}{\mathrm{gs}}_{i}{\mathrm{VPD}}_{i}-T_{\mathrm{cea},\;i}\right|$$

where $k_{T}$ is a learnable scaling coefficient. Rather than enforcing surface‐atmosphere energy balance closure, which is infrequently observed across the eddy covariance network (Mauder et al. 2024), the model accepts observed latent heat flux (LE) as the reference and encourages predicted gs to produce a physically plausible latent T, consistent with measurements. This loss ensures that gs predictions are realistic and constrained by observed fluxes, without directly correcting NEE or other carbon fluxes for energy imbalance.

We also add a nighttime GPP suppression loss function to penalize unphysical nighttime carbon uptake for ecosystems without CAM vegetation:

(5) $$L_{\text{night}}=\frac{1}{N}\sum_{i}^{N}\left(1-\mathrm{mas}k_{i}\right)\mathrm{GP}P_{i}$$

where (m_*i*_ = 1) if PPFD > 0.001 μmol m^−2^ s^−1^ and 0 otherwise.

The NEE loss terms are designed to ensure physical consistency of carbon closure.

(6) $$\mathrm{NE}E_{\text{pred}}={\text{RECO}}_{\text{KGML}}-{\mathrm{GPP}}_{\text{KGML}}$$

(7) $$L_{\mathrm{NEE}}=\frac{1}{N}\sum_{i}^{N}{\left(\mathrm{NE}E_{i}-\mathrm{NE}E_{\text{pred},i}\right)}^{2}$$

Physiological realism is further supported by losses on P_cea_ and R_cea_, which guide predictions (pred) toward partitioned flux observations (obs) without strictly enforcing them.

(8) $$P_{\mathrm{cea},\;\text{pred}}=\mathrm{GPP}-R_{\text{auto}}$$

(9) $$R_{\mathrm{cea},\;\text{pred}}=R_{\text{auto}-\text{below}}+R_{\mathrm{het}}$$

(10) $$L_{\text{Pcea}}=\frac{1}{N}\sum_{i}^{N}\left|P_{\mathrm{cea},\;\text{pred}}-P_{\mathrm{cea},\;\mathrm{obs}}\right|$$

(11) $$L_{\text{Rcea}}=\frac{1}{N}\sum_{i}^{N}\left|R_{\mathrm{cea},\;\text{pred}}-R_{\mathrm{cea},\;\mathrm{obs}}\right|$$

Moreover, a baseline RECO loss (L_RECO‐baseline_), derived from the mean nighttime NEE of the previous night (Trifunov et al. 2021), prevents the optimization from converging to trivial minimum‐magnitude solutions:

(12) $$L_{\text{RECO},\text{baseline}}=\frac{1}{N}\sum_{i}^{N}\text{ReLU}\left({\text{BASE}}_{\text{RECO}}-{\text{predicted}}_{\text{RECO}}\right)$$

where the rectified linear unit (ReLU) activation ensures the penalty is only applied when predicted RECO falls *below* the baseline, functioning as a one‐sided soft lower bound rather than a symmetric penalty. Finally, all loss functions are combined into a weighted objective function,

(13) $$L=\sum_{j}^{M}w_{j}L_{i}$$

where the weights $w_{j}$ were selected empirically to balance the contributions of each constraint and M is the number of all losses.

### 2.5 Experimental Design and Evaluation

We evaluated the KGML framework using a two‐step approach designed to assess physical consistency and to compare against conventional partitioning methods. Specifically, we assessed the WUE by comparing predicted GPP to T ratios with expected WUE values derived from environmental state variables, including PPFD, VPD, TA, and SM (see Table 1). We evaluated WUE distributions across biomes (IGBP classifications), WUE responses to environmental gradients (VPD and soil moisture), and diurnal WUE patterns to verify physiological consistency. We also evaluated the gs–PAR, gs‐VPD, and GPP‐T relationships with scatterplots and corresponding trend lines. Additionally, we evaluated carbon balance closure consistency, confirming that the predicted flux partitions satisfied the relationship NEE = RECO—GPP and maintained the correct sign and magnitude. Nighttime suppression of GPP under low‐light conditions (PPFD < 5 μmol m^−2^ s^−1^) was also evaluated to verify physiological plausibility. To validate overall model performance, we compared KGML‐predicted NEE (calculated as RECO—GPP) against eddy covariance measurements across all sites and time periods.

We then compared KGML predictions against conventional NT and DT partitioning methods across sites, plant functional types (PFTs), seasons, and light regimes (Figures 2 and 3). Statistical performance was assessed using RMSE, *R* ^2^, mean bias, and linear regression slopes. For PFT‐specific analysis, we evaluated diurnal cycles, seasonal trajectories (using 14‐day rolling means), annual totals, and method‐difference distributions separately for each IGBP class.

To investigate mechanistic differences between KGML and conventional partitioning methods, we analyzed the light‐dependent suppression of respiration (Kok effect) by examining RECO responses to low PPFD levels (0–600 μmol m^−2^ s^−1^). We binned data by PPFD intensity and compared diurnal patterns of RECO differences (KGML minus NT or DT) to identify when method divergence was greatest. Additionally, we decomposed KGML respiration predictions into R_a,above_, R_a,below_, and R_h_ components to examine their distinct temporal dynamics. Dawn (4:00–9:00 local standard time) and dusk (16:00–21:00) periods were analyzed separately to assess potential hysteresis in light‐respiration coupling.

Additionally, we evaluated predictive uncertainty of the KGML using Monte Carlo dropout (Gal and Ghahramani 2016) during inference, generating 90% confidence intervals (5th–95th percentiles) and comparing uncertainty width and structure against NT and DT bootstrap‐based uncertainties. We assessed whether KGML uncertainties exhibited appropriate heteroscedasticity (scaling with flux magnitude) and whether uncertainty envelopes varied systematically across diurnal and seasonal cycles.

Moreover, we assessed feature importance using SHapley Additive exPlanations (SHAP) analysis to quantify the contribution of individual input variables to model predictions (Lundberg and Lee 2017). This approach provides interpretable insights into how each environmental driver influences GPP and RECO estimates to understand the relative importance of the data‐driven components of the model (Losos et al. 2025; Ranjbar et al. 2025). We also evaluated the relative influence of each physical constraint using a gradient‐based importance analysis, which offers a computationally efficient proxy for assessing their contributions (Rodrigues et al. 2024; Wang et al. 2024). During training, the L2 norm of each physical loss term's gradient with respect to the model parameters was tracked across all batches, indicating how strongly each constraint guided model optimization (Zhang et al. 2024). The mean gradient magnitude for each term was then normalized to express its relative contribution as a percentage. This analysis is intended to provide an interpretable measure of the extent to which each physical rule (e.g., WUE, carbon mass balance, or sign conventions) influenced the learning dynamics, analogous to feature importance in explainable AI, but applied to physical regularization. Similar gradient‐based attribution techniques have been used in physics‐informed and constrained neural network frameworks to quantify the contribution of individual physical losses to model learning (Wang et al. 2024; Zhang et al. 2024). In addition, we performed systematic ablation experiments by sequentially removing individual physical constraints and retraining the model to quantify the degradation in predictive performance (*R* ^2^) attributable to each constraint.

## 3 Results

### 3.1 KGML Model Physical Constraints Fitness

**FIGURE 4:**
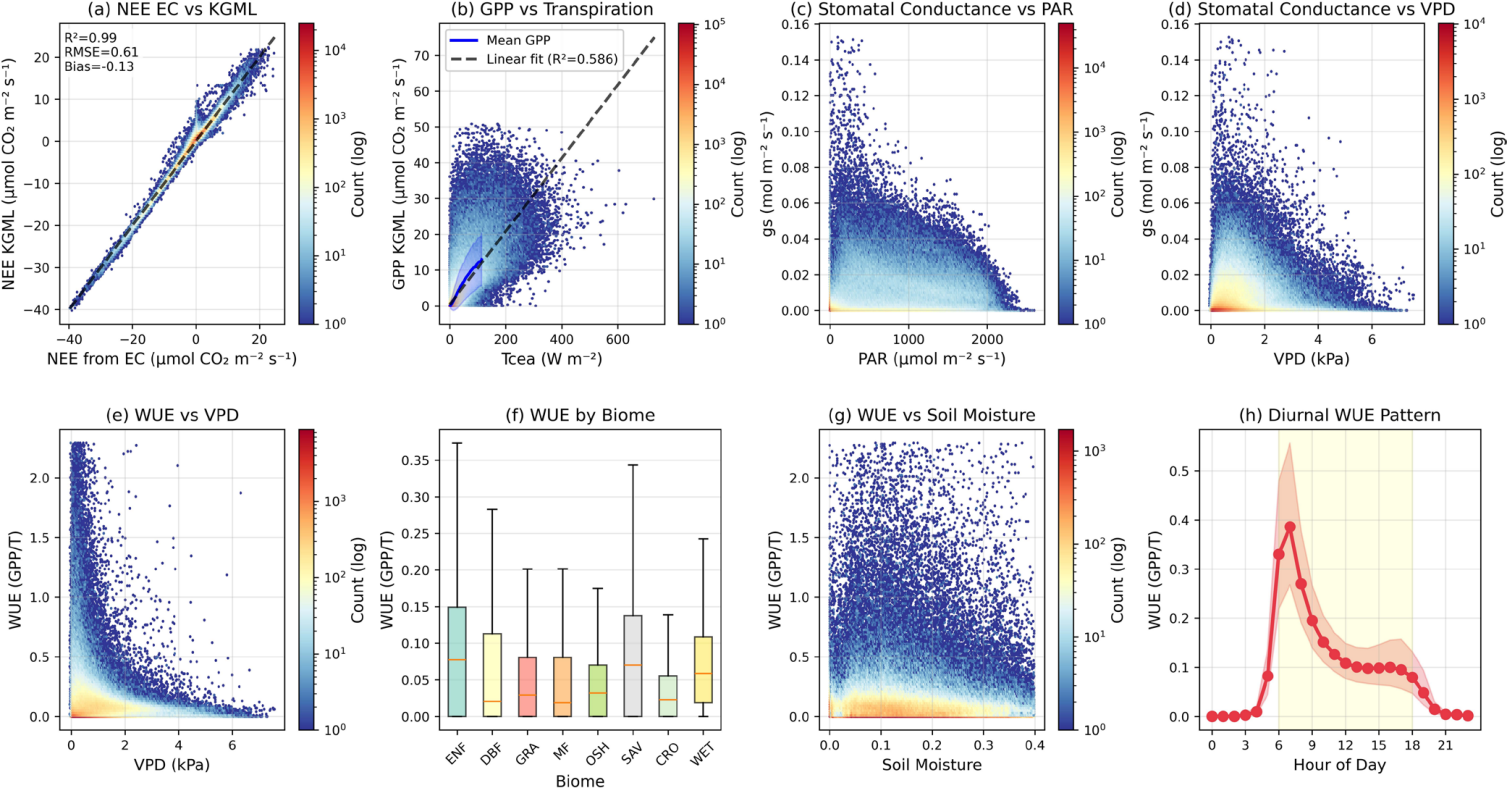
Physical soundness and mechanistic consistency of KGML carbon flux estimates. (a) NEE from eddy covariance versus KGML predictions (hexbin, log scale; 1:1 dashed line; *R* ^2^, RMSE, bias shown). (b) KGML GPP versus canopy transpiration (binned mean ± SD; linear fit). (c) gs versus PAR. (d) gs versus VPD (hexbin). (e) WUE (GPP/Transpiration, mmolCO_2_/H_2_O) versus VPD. (f) WUE distribution by IGBP biome (boxplots: Median, IQR, 1.5× IQR). (g) WUE versus soil moisture. (h) Diurnal WUE (5th–95th percentiles). Data are half‐hourly across NEON sites.

KGML‐modeled NEE closely matches eddy covariance measurements with *R* ^2^ = 0.99, RMSE of 0.61 μmol m^−2^ s^−1^, and a mean bias of 0.13 μmol m^−2^ s^−1^ (Figure 4a). The model captures key ecophysiological relationships: GPP shows a strong positive relationship with T (*R* ^2^ = 0.58; Figure 4b), consistent with coupled carbon‐water exchange. gs increases with PPFD across most of its range (Figure 4c) and exhibits the expected response to VPD, initially increasing at low VPD before declining at higher values (Figure 4d). WUE is constrained by VPD (Figure 4e), with distinct patterns across biome types (Figure 4f) and responses to soil moisture availability (Figure 4g). The diurnal pattern of WUE peaks before midday with values reaching approximately 0.4 mmolCO_2_/H_2_O on average (Figure 4h).

### 3.2 Differences Amongst Partitioning Methods

**FIGURE 5:**
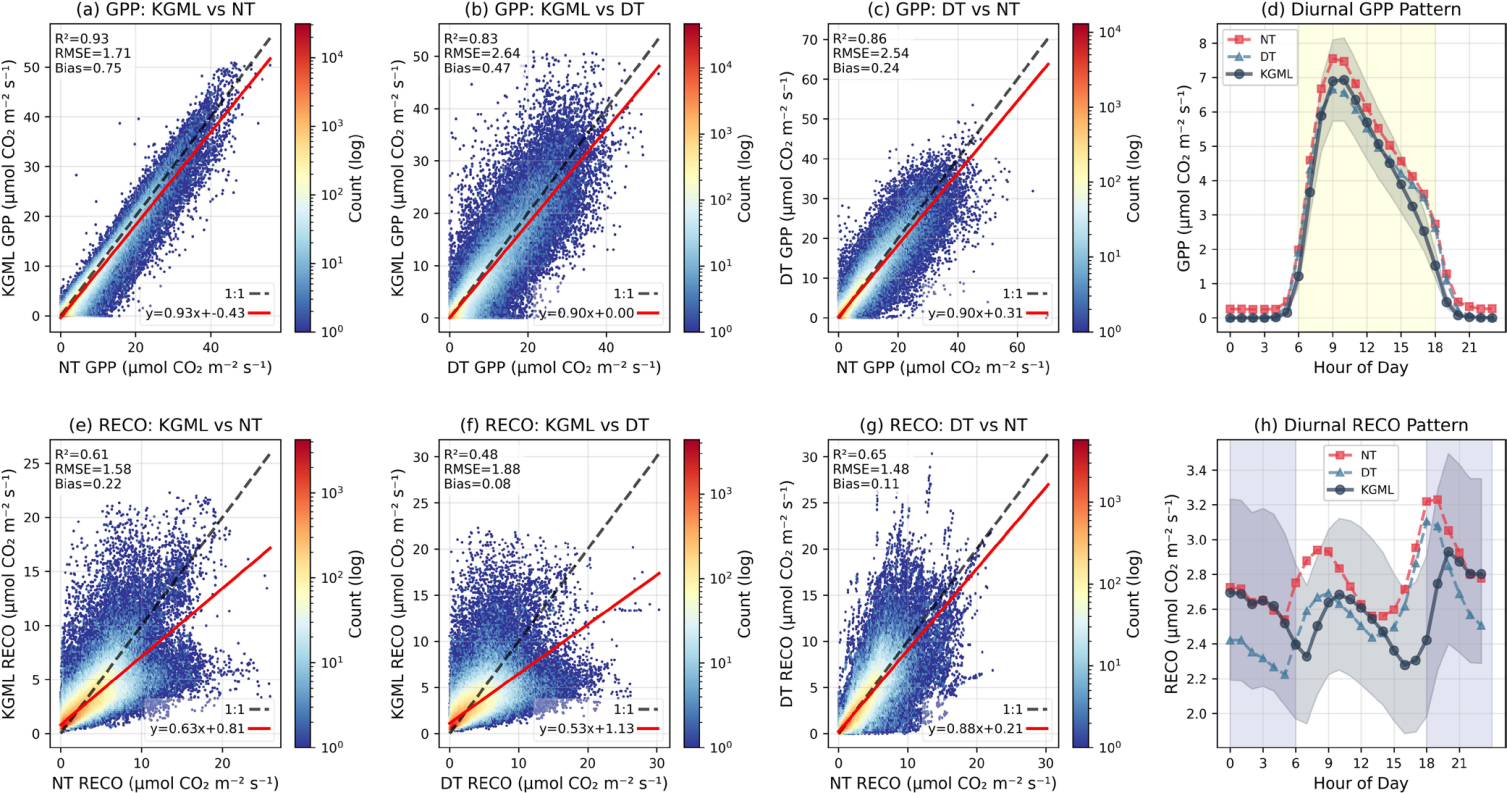
Comparative evaluation of KGML against NT and DT partitioning methods. Top row (a–d): GPP comparisons. (a) KGML versus NT. (b) KGML versus DT. (c) NT versus DT. Hexbin plots (log scale) with 1:1 dashed line, linear fit (red), and *R* ^2^, RMSE, bias. (d) Mean diurnal GPP for all methods with KGML 5th–95th percentile envelope. Bottom row (e–h): Same layout for RECO.

Comparison with conventional flux partitioning methods reveals both consistencies and systematic differences in KGML estimates. For GPP, KGML shows strong correlation with NT estimates (*R* ^2^ = 0.93, RMSE = 1.71 μmol m^−2^ s^−1^, bias = 0.75 μmol m^−2^ s^−1^) and slightly weaker agreement with DT estimates (*R* ^2^ = 0.83, RMSE = 2.64 μmol m^−2^ s^−1^, bias = 0.47 μmol m^−2^ s^−1^; Figure 5a,b). The diurnal patterns of KGML GPP predictions align well with both conventional methods, peaking around 10:00 local time, though KGML produces somewhat lower GPP magnitudes overall, especially in the afternoon (Figure 5d), although uncertainty bounds largely overlap. A key distinction is that KGML enforces a hard non‐negative constraint for GPP, unlike the conventional methods which occasionally produce negative values as the difference between measured NEE and the RECO model. For ecosystem respiration, KGML shows moderate correlation with both NT (*R* ^2^ = 0.61, RMSE = 1.58 μmol m^−2^ s^−1^, bias = 0.22 μmol m^−2^ s^−1^) and DT (*R* ^2^ = 0.48, RMSE = 1.88 μmol m^−2^ s^−1^, bias = 0.08 μmol m^−2^ s^−1^) estimates, with systematically lower magnitudes compared to conventional approaches (Figure 5e,f). The diurnal pattern of KGML RECO differs notably from conventional methods, showing a peak during late morning hours rather than the early morning peak characteristic of NT partitioning (Figure 5h).

**FIGURE 6:**
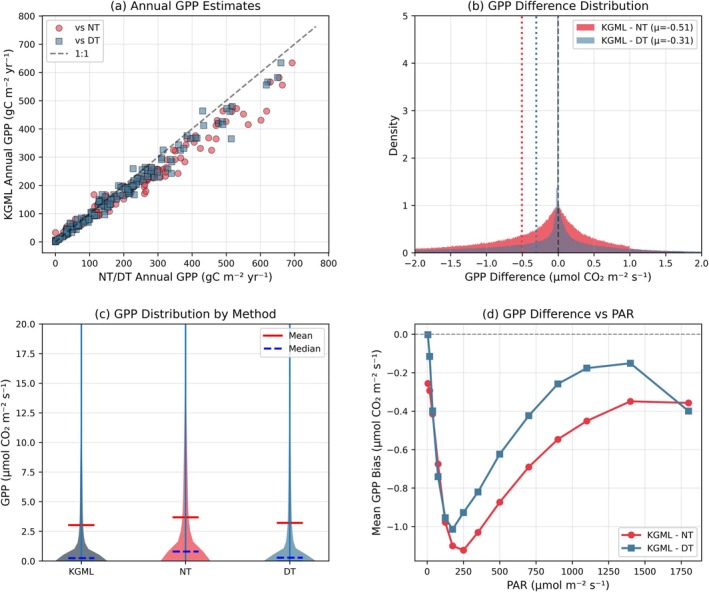
Distribution characteristics and systematic differences in GPP estimates across methods. (a) Annual GPP totals comparing KGML against NT (red circles) and DT (blue squares) methods across site‐years. The black dashed line the shows 1:1 relationship. (b) Probability density distributions of half‐hourly GPP differences (KGML minus NT in red; KGML minus DT in blue). Vertical dashed lines indicate mean bias; solid black line shows zero difference. (c) Violin plots comparing GPP distributions across methods. (d) Mean GPP bias (from NT/DT) as a function of light availability (PPFD bins). Gray dashed line indicates zero bias reference.

Annual GPP estimates from KGML are lower than both NT and DT methods across site‐years (Figure 6a). The relationship is approximately linear, though KGML estimates generally fall below the 1:1 line for both comparisons. At the half‐hourly scale, the distribution of GPP differences shows that KGML minus NT averages −0.51 μmol m^−2^ s^−1^, while KGML minus DT averages −0.31 μmol m^−2^ s^−1^ (Figure 6b). Both distributions are approximately normal and centered slightly below zero. The overall distribution of GPP values shows that KGML produces estimates with similar central tendency to NT and DT, though with slightly tighter spread (Figure 6c).

### 3.3 Light‐Dependent Patterns

The magnitude of GPP bias varies with light availability: at low PPFD (0–150 μmol m^−2^ s^−1^), KGML shows the largest negative bias relative to both NT (approximately −1.1 μmol m^−2^ s^−1^) and DT (approximately −1.0 μmol m^−2^ s^−1^). As PPFD increases, the bias diminishes, reaching approximately −0.5 μmol m^−2^ s^−1^ for NT and −0.2 μmol m^−2^ s^−1^ for DT at moderate light levels (750–1000 μmol m^−2^ s^−1^, Figure 6d).

**FIGURE 7:**
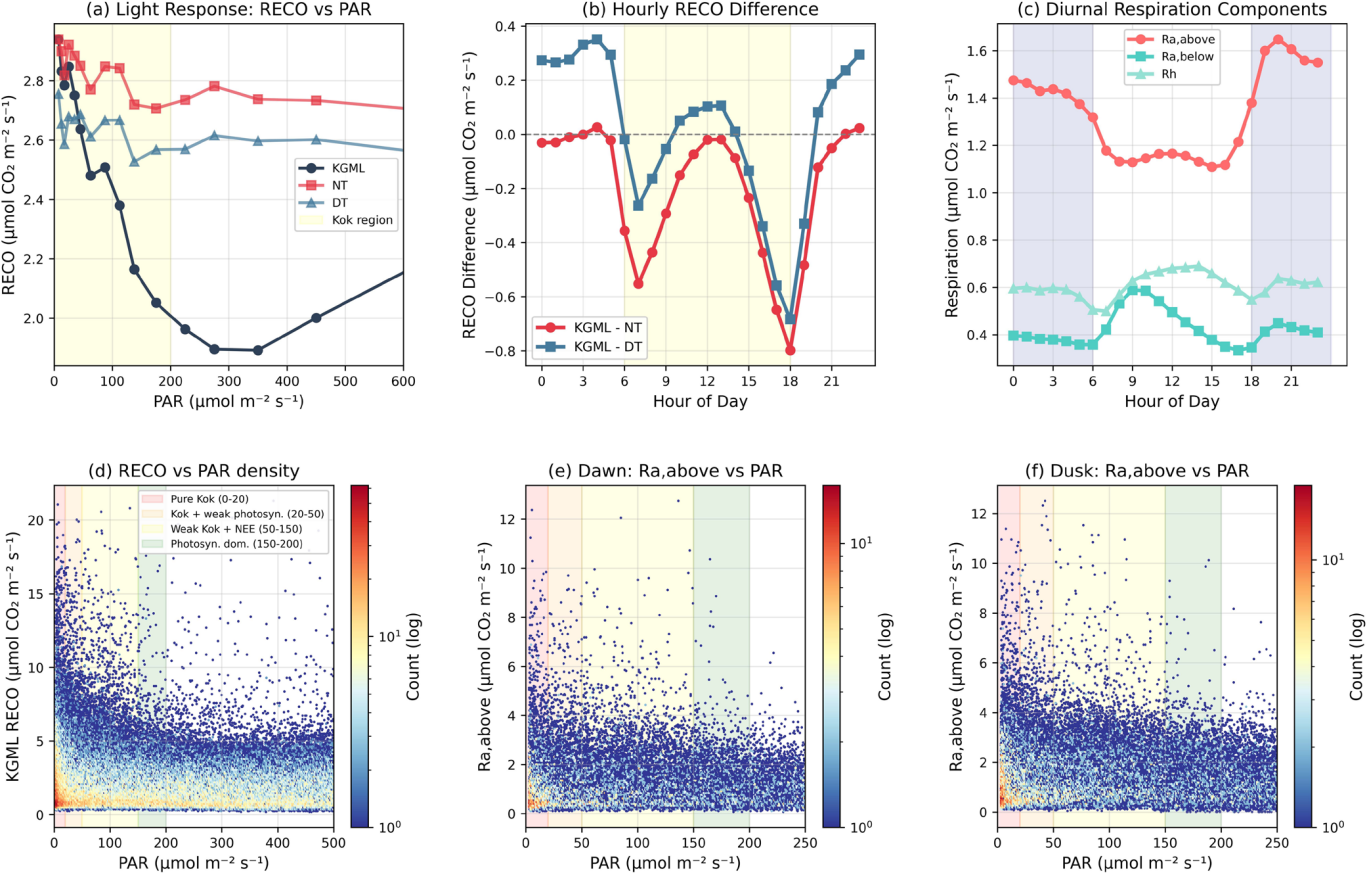
Mechanistic basis for method differences: Kok effect and light‐dependent respiration. (a) RECO versus PPFD (< 600 μmol m^−2^ s^−1^); yellow shading marks Kok‐sensitive region. (b) Diurnal RECO differences (KGML − NT or DT). (c) KGML respiration components: Above‐ground autotrophic (R_a,above_), below‐ground autotrophic (R_a,below_), and heterotrophic (Rh). (d) RECO versus PPFD (0–500 μmol m^−2^ s^−1^) with PPFD regimes color‐coded. (e) Dawn relationship between R_a,above_ and PAR. (f) Dusk relationship between R_a,above_ and PAR.

RECO estimates show distinct light‐dependent patterns across methods. In the low‐to‐moderate PPFD range (0–600 μmol m^−2^ s^−1^), KGML RECO exhibits a characteristic suppression pattern, declining from approximately 2.8 μmol m^−2^ s^−1^ at darkness to a minimum of approximately 1.9 μmol m^−2^ s^−1^ around 300 μmol m^−2^ s^−1^ PAR, before increasing again at higher light levels (Figure 7a). In contrast, both NT and DT methods show relatively stable or slightly declining RECO across this PPFD range. The region between 0 and 200 μmol m^−2^ s^−1^ PPFD (highlighted in yellow) corresponds to the Kok effect‐sensitive zone where light‐induced respiration suppression is expected (Fu et al. 2012; Gauthier et al. 2018; Heskel et al. 2013; Léger‐Daigle et al. 2022; Sharp et al. 1984). The diurnal pattern of RECO differences reveals time‐dependent divergence between methods (Figure 7b). KGML minus NT differences show negative values (approximately −0.4 to −0.5 μmol m^−2^ s^−1^) during early morning hours (5:00–9:00), reaching a minimum around hour 7:30, then increasing toward zero during midday, and becoming negative again (approximately −0.8 μmol m^−2^ s^−1^) during late afternoon/early evening hours (15:00–18:00). KGML minus DT differences follow a similar pattern but with different magnitudes, showing positive values during nighttime and early morning hours (0:00–5:00), negative values during mid‐morning to afternoon (approximately −0.3 to −0.4 μmol m^−2^ s^−1^), and returning to near‐zero or positive values in the evening. Decomposition of KGML respiration into component fluxes shows that R_a,above_ exhibits the most pronounced diurnal variation, peaking around 19:00 at approximately 1.65 μmol m^−2^ s^−1^, with lower values during nighttime (approximately 1.1–1.2 μmol m^−2^ s^−1^). R_a,below_ shows more moderate diurnal variation, ranging from approximately 0.35 to 0.6 μmol m^−2^ s^−1^ with peaks around 9:00 and 20:00, the latter broadly consistent with new findings that demonstrate that soil respiration is greater at night (Huang et al. 2026). R_h_ remains relatively stable across the day at approximately 0.6 μmol m^−2^ s^−1^ (Figure 7c). The relationship between KGML RECO and PPFD across different light regimes shows distinct density patterns (Figure 7d), with the highest data density occurring in the pure Kok region (0–20 μmol m^−2^ s^−1^, pink shading, Heskel et al. (2013); Sharp et al. (1984)) and the transition zone (20–50 μmol m^−2^ s^−1^, orange shading, Heskel et al. (2013); Yin et al. (2020)). During dawn hours, above‐ground autotrophic respiration shows minimal variation with increasing PPFD at low light levels (0–50 μmol m^−2^ s^−1^), which decline from approximately 3 to below 2 μmol m^−2^ s^−1^ as PPFD increases beyond 50 μmol m^−2^ s^−1^ (Figure 7e). During dusk hours, a similar pattern emerges, though with more scatter and slightly different magnitudes (Figure 7f).

### 3.4 KGML Carbon Flux Dynamics Across Plant Functional Types

**FIGURE 8:**
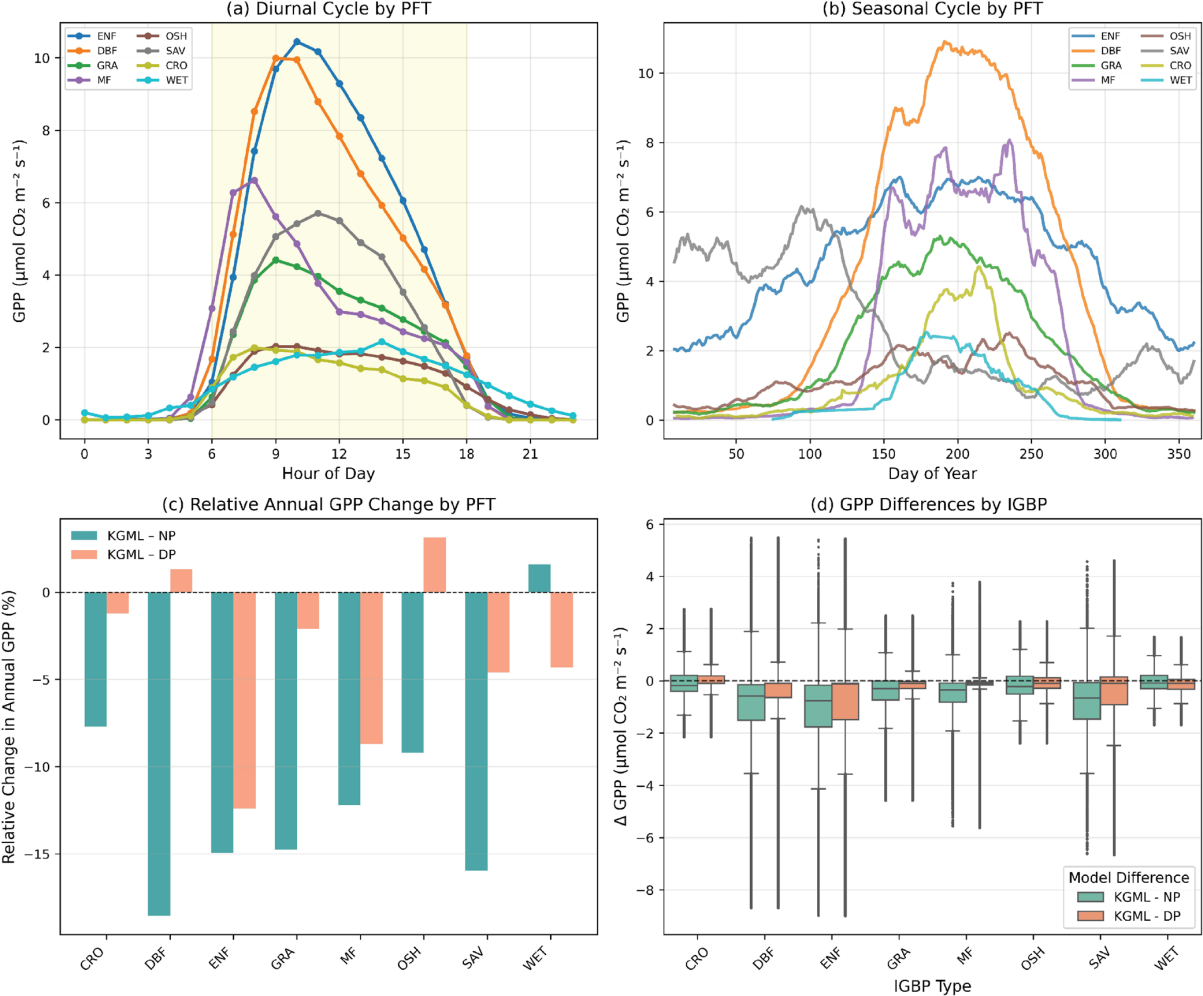
KGML GPP dynamics and method performance in different Plant functional types (PFT). (a) Mean diurnal GPP cycles by IGBP PFT. (b) Seasonal GPP trajectories by day of year using a 14‐day rolling mean. (c) relative changes in annual GPP by PFT. (d) Distributions of GPP differences between KGML and NT and DT partitioning methods by PFT, shown as boxplots truncated at the 1st–99th percentiles; dashed line denotes zero difference.

KGML GPP estimates show distinct patterns across plant functional types in their diurnal cycles and seasonal trajectories. The mean diurnal cycle varies substantially by PFT (Figure 8a), with evergreen needleleaf forests (ENF) and deciduous broadleaf forests (DBF) showing the highest peak GPP values, reaching approximately 10–11 μmol m^−2^ s^−1^ around 9:00–10:00. Grasslands (GRA) and open shrublands (OSH) exhibit intermediate peak values of approximately 5–6 μmol m^−2^ s^−1^ around hour 10:00–11:00. Savannas (SAV), croplands (CRO), and wetlands (WET) show the lowest peak values, ranging from approximately 2–4 μmol m^−2^ s^−1^. Mixed forests (MF) display intermediate behavior with peaks around 6–7 μmol m^−2^ s^−1^. All PFTs show near‐zero GPP during nighttime hours (taken here as approximately 0:00–6:00 and 18:00–24:00).

Seasonal GPP trajectories reveal diverse phenological patterns across PFTs (Figure 8b). Deciduous broadleaf forests show the most pronounced seasonal amplitude, with GPP rising sharply from near zero around day of year 100, peaking at approximately 11 μmol m^−2^ s^−1^ between days 150–200, and declining rapidly after day 250. Evergreen needleleaf forests display more sustained activity, beginning earlier in the year (day 50) at approximately 2–3 μmol m^−2^ s^−1^, reaching peaks of 6–7 μmol m^−2^ s^−1^ between days 150–200, and maintaining elevated values (4–5 μmol m^−2^ s^−1^) through day 250 before declining. Mixed forests show intermediate behavior with peak values around 8 μmol m^−2^ s^−1^. Grasslands and savannas exhibit moderate seasonal variation with peaks of approximately 4–5 μmol m^−2^ s^−1^ around days 200–220. Open shrublands show a distinctive pattern with relatively stable values throughout the growing season. Croplands and wetlands show lower seasonal amplitudes with peaks around 2–3 μmol m^−2^ s^−1^.

Relative annual GPP changes vary considerably across PFTs when comparing KGML against conventional partitioning methods (Figure 8c). For the nighttime partitioning approach (KGML—NT, shown in teal), most PFTs exhibit negative relative changes, indicating that KGML produces lower annual GPP estimates. The largest negative deviations occur in ENF and SAV, with relative changes around −15% to −18%. DBF, GRA, and MF show intermediate negative changes of approximately −8% to −12%. In contrast, WET showed a positive relative change of approximately +3%. For the daytime partitioning approach (KGML—DT, shown in coral), the pattern differs markedly. Two PFTs show positive relative changes, most notably OSH with approximately +3%–4%, and DBF with changes around +1%. Evergreen needleleaf forests show moderate negative changes of approximately −12%, while savannas and WET exhibit negative changes of approximately −4% to −5%.

The distribution of GPP differences between KGML and conventional partitioning methods shows PFT‐dependent patterns (Figure 8d). Box plots comparing KGML minus NT (teal) and KGML minus DT (coral), truncated at the 1st‐99th percentiles, reveal that median differences are close to zero across most PFTs, as indicated by the dashed zero line. However, the spread and quartile ranges vary. SAV, DBF, and ENF show the widest distributions, with interquartile ranges extending approximately ±1 to ±2 μmol m^−2^ s^−1^. For most PFTs, the median difference between KGML and both NT and DT methods falls within ±0.5 μmol m^−2^ s^−1^, though the full distribution (excluding outliers beyond the 99th percentile) can extend to ±4 to ±6 μmol m^−2^ s^−1^ for some PFTs. The distributions for KGML minus NT and KGML minus DT are generally similar within each PFT, though with slight differences in their quartile positions.

A detailed analysis of KGML‐derived RECO dynamics across plant functional types (PFTs) is provided in Text S1 and Figure S1. Briefly, KGML reveals distinct diurnal and seasonal RECO patterns among ecosystems, with deciduous broadleaf forests (DBF) showing the strongest temporal variability and croplands, wetlands, and open shrublands exhibiting comparatively lower respiration levels. Comparisons with conventional nighttime and daytime partitioning approaches further indicate that KGML generally produces lower annual RECO estimates across most PFTs, though differences vary by ecosystem type and method. The distribution of differences between KGML and conventional approaches also shows that median values remain close to zero for most PFTs, with variability differing among ecosystems. Full descriptions of the diurnal patterns, seasonal patterns, relative annual changes, and distributional differences are provided in Text S1, with supporting visualizations in Figure S1.

### 3.5 Physical Constraint and Feature Importance Analysis

**FIGURE 9:**
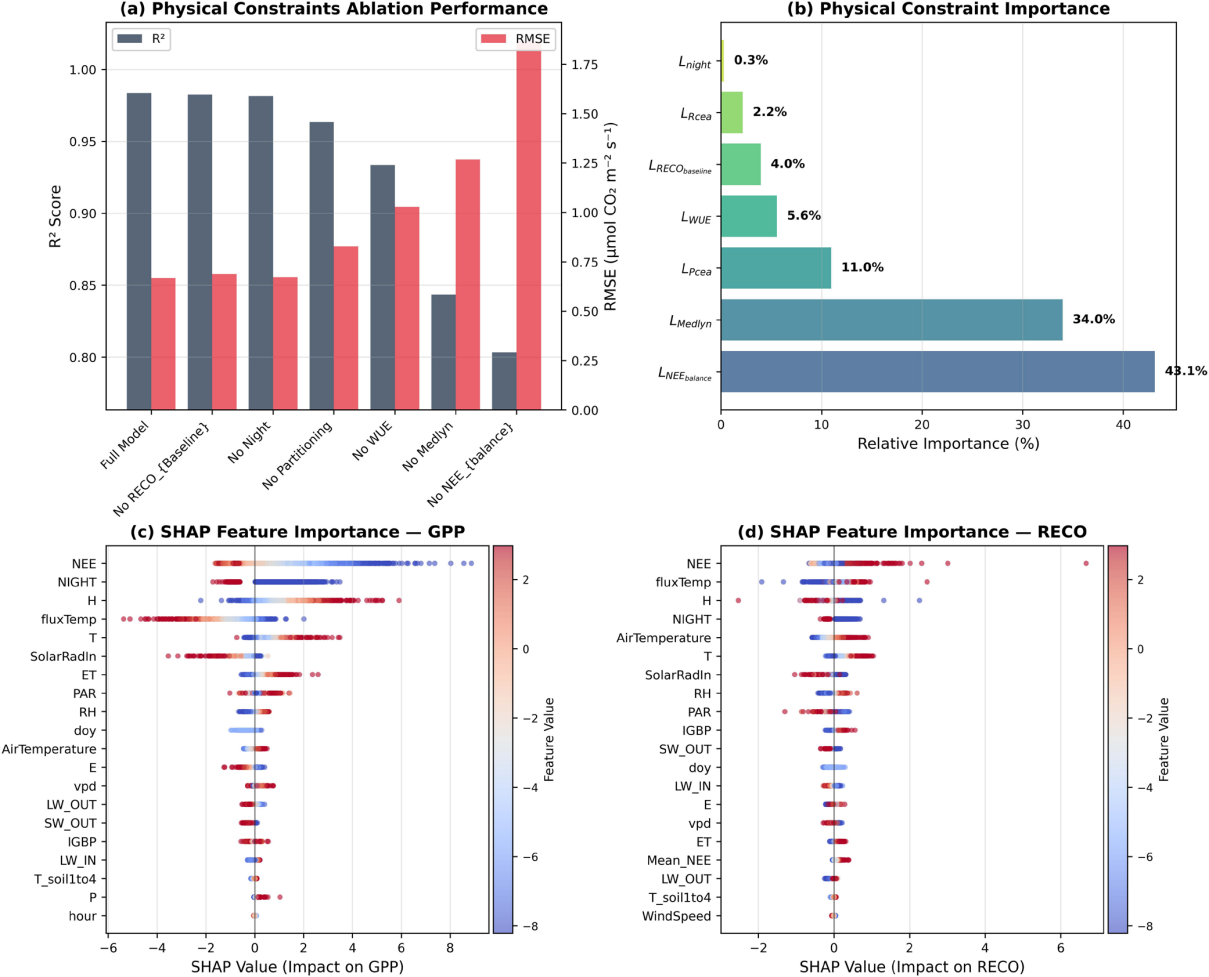
Physical constraints and feature importance in the KGML architecture. (a) Model performance (*R* ^2^) under systematic ablation of individual physical constraints. (b) Relative contribution of physical constraints quantified by batch‐wise L2 gradient norms during training, normalized to 100%. (c) SHAP feature importance for GPP prediction, showing mean absolute SHAP values across samples. (d) SHAP feature importance for RECO prediction.

Systematic ablation of individual physical constraints reveals their differential contribution to overall model performance (Figure 9a). The full KGML model achieves *R* ^2^ = 0.98 with RMSE of 0.65 μmol m^−2^ s^−1^. Removing the RECO baseline constraint (No RECO_Baseline) or the nighttime constraint (No Night) maintains similar *R* ^2^ (0.98) and RMSE (0.65 μmol m^−2^ s^−1^), indicating these constraints had minimal impact on carbon balance agreement (KGML derived NEE versus EC‐measured NEE). However, these constraints still play an important role in guiding the optimal solution of the model; for example, when removed, the model could converge toward reduced flux amplitude solutions, even though performance metrics remain similar. Ablating the partitioning constraint (No Partitioning) and WUE constraint (No WUE) causes a slight decrease to *R* ^2^ = 0.96 and *R* ^2^ = 0.93, respectively. The most severe performance loss occurs when the Medlyn stomatal conductance constraint (No Medlyn) or NEE balance constraint (No NEE Balance) is removed, resulting in *R* ^2^ = 0.84 or *R* ^2^ = 0.80, respectively.

Gradient‐based analysis quantifies the relative importance of physical constraints during model training (Figure 9b). The NEE balance constraint dominates with 43.1% of total gradient magnitude, indicating it exerts the strongest influence on parameter updates. The Medlyn stomatal optimality constraint accounts for 34.0% of gradient contributions, making it the second most influential constraint. The partitioned canopy exchange (P_cea_) contributes 11.0%, while the WUE constraint accounts for 5.6%. The RECO baseline constraint contributes 4.0%, followed by Rcea at 2.2% and the nighttime constraint at 0.3%. This distribution reveals that carbon balance (NEE) and stomatal optimization (Medlyn) constraints are the primary drivers of model learning dynamics, collectively accounting for over 77% of gradient influence.

SHAP feature importance analysis identifies the key drivers influencing GPP predictions (Figure 9c, see Table S2 for predictors descriptions). NEE (net ecosystem exchange) shows the strongest overall impact, with SHAP values distributed across a wide range, indicating both positive and negative influences depending on conditions. NIGHT exhibits predominantly negative SHAP values with high feature values shown in red. H (sensible heat flux), fluxTemp (sonic temperature) and T, all related to the thermal environment, display moderate impacts. For RECO predictions, SHAP analysis reveals a distinct set of influential features (Figure 9d). NEE again shows the broadest SHAP value distribution, demonstrating its importance across both GPP and RECO estimation. NIGHT exhibits primarily negative SHAP values. AirTemperature, T, and SolarRadin show moderate impacts with SHAP values generally within ±2.

## 4 Discussion

### 4.1 KGML Performance: Physical Consistency

The relationships shown in Figure 4 indicate that the KGML model successfully partitioned NEE into GPP and RECO components while maintaining adherence to the defined physical and biological constraints. The model effectively captures ecophysiological patterns consistent with established theoretical understanding (Hatfield and Dold 2019; Ito and Inatomi 2012; Manzoni et al. 2011). The strong constraint of WUE by VPD arises because increasing VPD drives higher atmospheric demand for water, prompting partial stomatal closure to prevent excessive water loss, which in turn limits photosynthesis (Hatfield and Dold 2019). This negative relationship is widely observed across ecosystems and aligns with optimal stomatal function theories that balance carbon gain with water conservation (Katul et al. 2010; Medlyn et al. 2011; Wang et al. 2021). The decline of gs at high VPD is again consistent with physiological regulation, where stomatal closure occurs to prevent hydraulic stress under dry atmospheric conditions (Grossiord et al. 2020; López et al. 2021).

The monotonic increase in GPP with T indicates that the model effectively captured the linkage between photosynthetic carbon assimilation and transpiration‐driven energy fluxes, consistent with the coupled behavior of carbon and water cycles regulated by stomatal processes (Gentine et al. 2019; Ito and Inatomi 2012), noting that these can become ‘decoupled’ at high temperatures (De Kauwe et al. 2019; Schuler et al. 2025; Xia et al. 2025) and the two fluxes have different optimal temperatures (Xia et al. 2025). The near‐perfect carbon balance relationship (*R* ^2^ = 0.99) confirms that the physical constraints embedded in the model enforce coherence among NEE, GPP, and RECO, avoiding the imbalances that can occur in unconstrained or purely empirical models (Karpatne et al. 2024). This high level of carbon balance closure is important because it demonstrates that the framework avoids the underconstrained inverse problem, where multiple (GPP, RECO) combinations could fit NEE. The combination of carbon mass balance enforcement (43.1% of gradient contribution), Medlyn stomatal optimality (34.0%), and ancillary constraints (WUE, RECO baseline, partitioning guidance) collectively stabilize flux magnitudes and prevent convergence toward low‐amplitude solutions. The ablation experiments (Figure 9a) further demonstrate that no single constraint dominates the solution; rather, accurate flux magnitudes emerge from the synergistic interaction of multiple physical rules.

### 4.2 Method Differences and Reasons: Light‐Dependent Patterns Analysis

The systematic differences between KGML and conventional partitioning methods revealed in Figures 5, 6, 7 reflect the incorporation of coupled physiological constraints that account for processes often neglected in empirical approaches. The strong correlations with both NT (*R* ^2^ = 0.93) and DT (*R* ^2^ = 0.83) methods demonstrate that KGML captures the overall temporal dynamics and magnitudes of carbon fluxes, while the systematic differences, particularly the light‐dependent patterns shown in Figure 6d, suggest that the framework resolves, or attempts to resolve, additional physiological mechanisms.

The largest negative GPP difference at low PPFD (0–150 μmol m^−2^ s^−1^; approximately −1.0 to −1.1 μmol m^−2^ s^−1^ relative to both NT and DT) coincides with transition periods where multiple physiological processes interact. NT methods typically assume that respiration rates derived from nighttime conditions are directly applicable to daytime periods (Whitehead et al. 2004). However, multiple lines of evidence suggest this assumption may not hold. First, leaf‐ and canopy‐level studies have documented light‐induced suppression of mitochondrial respiration, known as the “Kok effect,” which can reduce daytime respiration by 10%–20% (Heskel et al. 2013; Keenan et al. 2019; Kok 1949; Tramontana et al. 2020; Yin et al. 2020). Second, stomatal‐transpiration coupling becomes particularly important during low‐light transitions, when partial stomatal closure limits both CO_2_ uptake and water loss (Gentine et al. 2019). Third, the allocation between autotrophic and heterotrophic respiration components may shift diurnally in response to canopy microclimate and substrate availability (Janssens et al. 2001).

The RECO suppression pattern shown in Figure 7a is qualitatively consistent with the Kok effect, and the timing of this suppression aligns with the low‐light zone (0–200 μmol m^−2^ s^−1^) where light inhibition is expected to be most pronounced. However, we emphasize that this pattern is not uniform across all ecosystems or measurement periods. The diurnal analysis (Figure 7b) reveals that the magnitude and timing of RECO differences vary substantially throughout the day, with negative deviations from NT methods peaking during morning hours (approximately −0.4 to −0.5 μmol m^−2^ s^−1^ around 7:30) and again during late afternoon (approximately −0.8 μmol m^−2^ s^−1^ between 15:00 and 18:00) when air and soil temperatures are relatively high and temperature‐based RECO models peak. This behavior may indicate that the KGML framework provides a better daytime RECO estimate, as NT temperature‐based models can become overly sensitive to elevated afternoon temperatures, potentially leading to overestimation of respiration during warm periods, which was a motivation for the VPD limitation in the DT method (Lasslop et al. 2010).

Importantly, the decomposition of RECO into component fluxes (Figure 7c) reveals that R_a,above_ exhibits the most pronounced diurnal variation. This pattern differs from the early morning peak typically observed in conventional NT‐partitioned RECO. The early morning peak in NT methods may reflect both respiratory activity and transport dynamics: as turbulence increases after sunrise, stored CO_2_ from nighttime respiration within the canopy and near‐surface layers can be rapidly released, producing a transient flux peak that reflects ventilation rather than instantaneous ecosystem respiration (Aubinet et al. 2012). This effect is likely more pronounced in dense canopies, where CO_2_ accumulation overnight is often greater than in more open ecosystems.

The dawn and dusk analyses (Figure 7e,f) further support the presence of light‐dependent respiration modulation. R_a,above_ shows minimal variation at very low PPFD (0–50 μmol m^−2^ s^−1^), consistent with the pre‐illumination phase, then declines as PPFD increases beyond 50 μmol m^−2^ s^−1^. This transition zone corresponds to the onset of photosynthetic activity and the potential initiation of Kok‐effect‐like suppression. However, we acknowledge that the scatter in these relationships and the ecosystem‐dependent variability (Figure 8) indicate that no single mechanistic explanation fully accounts for the observed patterns.

Based on the analyses and the integration of GPP_DT_ as a consistent reference, we conclude that the reduced GPP and RECO inferred by KGML likely reflects a combination of mechanisms: (i) partial light‐induced suppression of daytime respiration, particularly in forested ecosystems where the effect has been best documented (Keenan et al. 2019; Yin et al. 2020); (ii) stomatal regulation linking transpiration and photosynthesis, where partial closure under stress (e.g., high VPD or low soil moisture) limits CO_2_ uptake relative to water vapor flux (Gentine et al. 2019); (iii) dynamic allocation between autotrophic and heterotrophic respiration components in response to environmental drivers; and (iv) residual model structural uncertainty. While we observe patterns qualitatively consistent with the Kok effect, particularly the PPFD‐dependent suppression in the 0–300 μmol m^−2^ s^−1^ range and the diurnal timing of RECO differences, we do not claim that this is the sole explanation. Rather, the KGML framework integrates multiple coupled constraints, and the emergent flux patterns reflect the optimal balance among multiple physical rules embodied in the model structure and biological constraints embodied in the loss functions.

### 4.3 KGML Partitioning of Carbon Fluxes Across Plant Functional Types

The consistent seasonal evolution of GPP across all PFTs shows that KGML effectively captures large‐scale phenological dynamics governed by radiation and temperature. The agreement in seasonal timing with conventional methods indicates that physical and biological constraints did not distort temporal patterns but refined flux magnitudes to remain within physiological boundaries. The negative annual GPP differences for most forest types (ENF: −15% to −18% vs. NT; DBF: −8 to −12% vs. NT; Figure 8c) are consistent with expectations for dense canopies, where stronger autotrophic respiration and potential light‐induced respiration suppression can reduce apparent gross uptake during the day (Harmon et al. 2011; Piao et al. 2010). Conventional NT approaches often neglect these effects, potentially inflating daytime GPP estimates, especially in forest ecosystems with more biomass to respire.

In contrast, the positive GPP differences observed in some non‐forest PFTs, most notably OSH (+3%–4% vs. DT) and WET (+3% vs. NT), indicate that KGML infers higher productivity in these systems under certain conditions. For open shrublands, this pattern may reflect enhanced light penetration and greater water‐use efficiency, leading to stronger radiation‐carbon coupling (Du et al. 2022; Emmerich 2007; Reich et al. 2026). Interestingly, this contrasts with some prior analyses where open shrublands showed lower GPP when accounting for light‐induced respiration suppression (Keenan et al. 2019), suggesting that the muted Kok effect in these ecosystems may arise from relatively low aboveground respiration available to be suppressed.

The corresponding RECO analysis (Figure S1) reveals complementary patterns. Forest ecosystems show higher absolute RECO values due to larger biomass pools driving respiration. Open shrublands and croplands, despite lower biomass, show relatively higher RECO in the KGML model to maintain carbon mass closure. These ecosystems experience stronger fluctuations in environmental drivers and have faster turnover of surface organic material (Austin and Vivanco 2006), which increases modeled respiration relative to forests when physiological constraints are enforced.

There is no single mechanistic explanation for KGML flux differences; rather, the framework integrates multiple physiological constraints (stomatal regulation, WUE optimization, respiration partitioning, carbon mass balance), and the emergent patterns reflect ecosystem‐specific balances among these competing rules. The tighter distributions and near‐zero median differences shown in Figure 8d indicate that, at the half‐hourly scale, KGML and conventional methods agree well on average, with discrepancies primarily reflecting the treatment of edge cases (low light including nighttime transitions and high VPD) where physiological constraints are most consequential.

### 4.4 Physical Constraint and Feature Importance Analysis

The ablation experiments and gradient analyses (Figure 9) provide mechanistic insight into how the KGML framework achieves its flux estimates and why it differs from conventional methods. The dominance of the NEE balance constraint (43.1% of gradient contribution) is expected, as NEE directly governs the partitioning of carbon fluxes and serves as the fundamental boundary condition preventing non‐physical solutions (Karpatne et al. 2024; Liu et al. 2024). Importantly, the strong influence of the Medlyn stomatal optimality constraint (34.0% of gradient contribution) indicates that stomatal regulation serves as the primary physiological mechanism guiding the model's internal representation of plant carbon‐water exchange. This rule directly links stomatal conductance to VPD and photosynthetic demand, shaping the model's sensitivity to environmental drivers (Medlyn et al. 2011; Sabot et al. 2022). Because this constraint inherently enforces water and carbon flux coupling, it likely subsumes some of the influence of other related physical rules, such as WUE (5.6%), which becomes less influential once the Medlyn model loss function guides the solution to simulate typical stomatal behavior.

The relatively modest contribution of the RECO baseline constraint (4.0%) is noteworthy. While this constraint has minimal impact on NEE reproduction (*R* ^2^ remains 0.98 when ablated; Figure 9a), it plays a critical role in preventing the low‐amplitude solutions that can arise in underconstrained inverse problems. By penalizing daytime RECO values that fall below a certain level of the nocturnal baseline, this constraint guides respiration magnitudes without over‐restricting diurnal variability. The low gradient contribution suggests that, during training, the model quickly learns to satisfy this lower bound, after which the constraint exerts minimal additional influence. The nighttime constraint (0.3% of gradient contribution) serves primarily as a regularization term rather than a primary driver of behavior. By enforcing zero GPP during darkness, it prevents physically implausible solutions and improves model interpretability, but the low gradient contribution indicates that the model would naturally approach this behavior even without explicit enforcement, likely due to the strong correlation between PPFD and photosynthetic activity captured by the data.

The SHAP analysis (Figure 9c,d) complements the gradient‐based results by revealing which environmental variables drive flux predictions at the sample level. For GPP, the dominance of NEE reflects the fact that observed net flux provides strong information about gross uptake, particularly when combined with respiration estimates. The importance of night‐time masking (NIGHT), sensible heat flux (H), temperature, and partitioned transpiration (T) indicates that the model integrates multiple environmental signals, especially those related to light and temperature to infer photosynthetic activity. For RECO, similar energy flux drivers and temperature variables are most important, consistent with the temperature sensitivity of respiratory processes (Hursh et al. 2017; Niu et al. 2024). The overlap in key features for both fluxes indicates that KGML captures shared environmental controls (Austin and Vivanco 2006; Baldocchi et al. 2015), while differentiating between photosynthetic and respiratory responses through embedded physical constraints.

Together, these analyses demonstrate that the KGML framework achieves physically consistent flux estimates through the synergistic interaction of multiple constraints, with carbon mass balance and stomatal optimization serving as the primary organizing principles. Secondary constraints (WUE, RECO baseline, nighttime masking) serve largely as regularization terms that prevent overfitting and maintain physiological plausibility. This multi‐constraint design reduces underconstrained inverse problems by enforcing multiple independent physical relationships, preventing convergence to low‐magnitude or degenerate solutions.

### 4.5 Limitations and Future Work

KGML improves upon traditional flux partitioning by integrating key physical rules and biology‐based relationships into a self‐supervised framework that blends mechanistic understanding with data‐driven flexibility. Its transformer‐based architecture, still relatively uncommon in eddy covariance research (Lucarini et al. 2024), captures temporal dependencies while the Monte Carlo dropout calibrates uncertainty estimates without explicit statistical assumptions (Figure S8). However, several limitations warrant consideration. First, the model's reliance on partitioned carbon and water components (E, T, P, and R derived from cea) ties its accuracy to input quality. While supplementary fvs‐based analysis provides independent validation (Figures S2–S7), full characterization of uncertainties in these upstream products (Zahn and Bou‐Zeid 2024a) remains beyond the current scope. Future work incorporating independent measurements, such as isotopic partitioning, chamber‐based respiration, or sap flow data, would help isolate model performance from input data quality. Second, the systematic differences between KGML and conventional methods, while consistent with known physiological processes, cannot be fully validated without independent reference data for partitioned fluxes. Direct measurements using isotopic or chamber methods, or GPP constraints from SIF retrievals, accounting for uncertainties in each, would help confirm whether KGML estimates are more accurate or simply internally consistent with imposed constraints.

Third, generalization beyond NEON sites may require retraining and necessitates high‐frequency (10–20 Hz) flux measurements for water vapor partitioning. The framework was developed exclusively on temperate and subtropical U.S. ecosystems, and performance in tropical, boreal, or Mediterranean systems remains uncertain. Finally, while the transformer architecture can handle temporal context, the current implementation treats each timestep independently and does not fully exploit carry‐over effects such as antecedent moisture influencing respiration; future efforts could incorporate additional ecological mechanisms including ‘memory’ effects (Liu et al. 2019).

Despite these limitations, the KGML framework demonstrates that integrating multiple physiological constraints within a flexible machine learning architecture can yield carbon flux estimates that are simultaneously data‐consistent and mechanistically plausible. The approach offers a pathway forward that neither relies purely on empirical correlations nor requires the full complexity of process‐based ecosystem models. As flux tower networks expand and ancillary data streams become more widely available, hybrid frameworks like KGML will be well‐positioned to synthesize diverse constraints into increasingly robust flux estimates.

## 5 Conclusion

This study presents a self‐supervised, KGML that partitions eddy covariance observations of net ecosystem exchange into GPP and RECO under explicit physical and biological constraints. By integrating hard physical constraints, mass balance, non‐negativity of fluxes, and zero nighttime GPP, with soft ecophysiological principles, including stomatal regulation, water‐use efficiency, and vapor pressure deficit responses, the KGML model combines mechanistic understanding with data‐driven flexibility. The framework achieves near‐perfect carbon balance closure (NEE = RECO‐GPP, *R* ^2^ = 0.99) while maintaining realistic representations of coupled carbon‐water exchange processes, demonstrating that multiple physiological constraints working in concert prevent the underconstrained inverse problems that can plague purely empirical partitioning approaches. At most sites, the model predicts lower GPP and RECO than conventional nighttime partitioning methods, with the largest differences occurring at low light levels and during transition periods. These systematic differences likely reflect multiple interacting mechanisms: light‐induced suppression of daytime respiration (qualitatively consistent with the Kok effect), stomatal‐transpiration coupling that limits carbon uptake under suboptimal conditions, dynamic allocation between respiration components, and explicit enforcement of non‐negativity constraints. We emphasize that no single mechanism fully explains the observed flux patterns; rather, emergent patterns reflect ecosystem‐specific balances among competing physical rules. The plant functional type analysis showed forests as the larger negative deviations (DBF: −8% to −12%; ENF: −15% to −18% vs. NT). The ablation experiments demonstrate that accurate flux magnitudes emerge from synergistic interaction of multiple constraints, with carbon mass balance (43.1%) and Medlyn stomatal optimality (34.0%) serving as primary organizing principles, while secondary constraints provide essential regularization.

Several limitations warrant consideration. The framework relies on partitioned carbon and water flux components whose uncertainties propagate into final estimates, though supplementary analyses using alternative methods yield qualitatively similar results. Systematic differences between KGML and conventional methods cannot be fully validated without independent reference data for partitioned fluxes, and generalization beyond NEON sites may require retraining. Despite these limitations, the KGML framework demonstrates that integrating multiple physiological constraints within a flexible machine learning architecture can yield carbon flux estimates that are simultaneously data‐consistent and mechanistically plausible. As flux tower networks expand and ancillary data streams, including isotopic measurements, solar‐induced fluorescence, and chamber‐based respiration data, become more widely available, hybrid frameworks like KGML will be well‐positioned to synthesize diverse constraints into increasingly robust flux estimates. This approach offers a pathway forward that neither relies purely on empirical correlations nor requires the full complexity of process‐based ecosystem models, instead leveraging the complementary strengths of mechanistic understanding and data‐driven learning.

## Author Contributions

**Sadegh Ranjbar:** conceptualization, data curation, formal analysis, investigation, methodology, software, validation, visualization, writing – original draft, writing – review and editing. **Ankur R. Desai:** conceptualization, funding acquisition, project administration, resources, supervision, writing – review and editing. **Sophie Hoffman:** data curation, methodology, visualization, writing – review and editing. **Einara Zahn:** conceptualization, data curation, methodology, software, writing – review and editing. **Elie Bou‐Zeid:** conceptualization, data curation, methodology, software, writing – review and editing. **Paul C. Stoy:** data curation, funding acquisition, investigation, project administration, resources, supervision, writing – original draft, writing – review and editing.

## Funding

This work was supported by the U.S. National Science Foundation, 2422397.

## Conflicts of Interest

The authors declare no conflicts of interest.

## Supporting information


**Figure S1:** KGML RECO dynamics and method performance in different Plant functional types (PFT). (a) Mean diurnal RECO cycles by IGBP PFT. (b) Seasonal RECO trajectories by day of year using a 14‐day rolling mean. (c) Annual RECO totals (gC m^−2^ yr.^−1^; mean ± SD) by PFT across site‐years; tropical/subtropical PFTs are excluded due to limited NEON coverage. (d) Distributions of RECO differences between KGML and nighttime (NT) and daytime (DT) partitioning methods by PFT, shown as boxplots truncated at the 1st–99th percentiles; the dashed line denotes zero difference. CEA partitioning data are used for this analysis. The cea estimates are used in the modeling part for this analysis.
**Figure S2:** Physical soundness and mechanistic consistency of KGML carbon flux estimates. (a) NEE from eddy covariance versus KGML predictions (hexbin, log scale; 1:1 dashed line; *R* ^2^, RMSE, bias shown). (b) KGML GPP versus canopy transpiration (binned mean ± SD; linear fit). (c) Stomatal conductance (gs) versus PAR. (d) gs versus VPD (hexbin). (e) WUE (GPP/Transpiration, mmolCO_2_/H_2_O) versus VPD. (f) WUE distribution by IGBP biome (boxplots: median, IQR, 1.5× IQR). (g) WUE versus soil moisture. (h) Diurnal WUE (5th–95th percentiles). Data are half‐hourly across NEON sites. The fvs estimates are used in the modeling part for this analysis.
**Figure S3:** Comparative evaluation of KGML against neural network and decision tree partitioning methods. Top row (a–d): GPP comparisons. (a) KGML versus nighttime (NT) neural network. (b) KGML versus daytime (DT) decision tree. (c) NT versus DT. Hexbin plots (log scale) with 1:1 dashed line, linear fit (red), and *R* ^2^, RMSE, bias. (d) Mean diurnal GPP for all methods with KGML 5th–95th percentile envelope. Bottom row (e–h): Same layout for RECO. Markers: circles (KGML), squares (NT), triangles (DT). The fvs estimates are used in the modeling part for this analysis.
**Figure S4:** The distributions and differences in GPP estimates across methods. (a) Annual GPP totals comparing KGML against NT (red circles) and DT (blue squares) methods across site‐years. The black dashed line shows 1:1 relationship. (b) Probability density distributions of half‐hourly GPP differences (KGML minus NT in red; KGML minus DT in blue). Vertical dashed lines indicate mean bias; the solid black line shows zero difference. (c) Violin plots comparing GPP distributions across methods. (d) Mean GPP bias (from NT/DT) as a function of light availability (PAR bins). Gray dashed line indicates zero bias reference. The fvs estimates are used in the modeling part for this analysis.
**Figure S5:** Mechanistic basis for method differences: Kok effect and light‐dependent respiration. (a) RECO versus PAR (< 600 μmol m^−2^ s^−1^); yellow shading marks the Kok‐sensitive region. (b) Diurnal RECO differences (KGML−NT/DT). (c) KGML respiration components: above‐ground autotrophic (R_a,above_), below‐ground autotrophic (R_a,below_), and heterotrophic (Rh). (d) RECO versus PAR (0–500 μmol m−2 s−1) with PAR regimes color‐coded. (e) Dawn relationship between R_a,above_ and PAR. (f) Dusk relationship between R_a,above_ and PAR. The fvs estimates are used in the modeling part for this analysis.
**Figure S6:** KGML GPP dynamics and method performance across different plant functional types (PFTs). (a) Mean diurnal GPP cycles by IGBP PFT. (b) Seasonal GPP trajectories by day of year using a 14‐day rolling mean. (c) relative changes in annual GPP by PFT. (d) Distributions of GPP differences between KGML and nighttime (NT) and daytime (DT) partitioning methods by PFT, shown as boxplots truncated at the 1st–99th percentiles; dashed line denotes zero difference. The fvs estimates are used in the modeling part for this analysis.
**Figure S7:** KGML RECO dynamics and method performance in different Plant functional types (PFT). (a) Mean diurnal RECO cycles by IGBP PFT. (b) Seasonal RECO trajectories by day of year using a 14‐day rolling mean. (c) Annual RECO totals (gC m^−2^ yr.^−1^; mean ± SD) by PFT across site‐years; tropical/subtropical PFTs excluded due to limited NEON coverage. (d) Distributions of RECO differences between KGML and nighttime (NT) and daytime (DT) partitioning methods by PFT, shown as boxplots truncated at the 1st–99th percentiles; dashed line denotes zero difference. CEA partitioning data are used for this analysis. The fvs estimates are used in the modeling part for this analysis.
**Figure S8:** Uncertainty quantification and propagation in KGML versus empirical methods. (a) Distribution of uncertainty width (95th–5th percentile) across methods; KGML from Bayesian ensemble and NT/DT from bootstrap resampling. Boxplots show median, IQR, and 1.5 × IQR whiskers. (b) Probability density of uncertainty widths with vertical dashed lines indicating means. (c) Diurnal GPP mean ±90% confidence intervals for KGML and NT. (d) KGML uncertainty (half‐width of 90% CI) versus flux magnitude with binned mean shown in red. The cea estimates are used in the modeling part for this analysis.
**Figure S9:** KGML carbon flux estimates for hold‐out test samples. (a) NEE from eddy covariance versus KGML predictions (hexbin, log scale; 1:1 dashed line; *R* ^2^, RMSE, bias shown). (b) KGML GPP versus canopy transpiration (binned mean ± SD; linear fit). (c) Stomatal conductance (gs) versus PAR. (d) gs versus VPD (hexbin). (e) WUE (GPP/Transpiration, mmolCO_2_/H_2_O) versus VPD. (f) WUE distribution by IGBP biome (boxplots: median, IQR, 1.5× IQR). (g) WUE versus soil moisture. (h) Diurnal WUE (5th–95th percentiles). Data are half‐hourly across NEON sites. The cea estimates are used in the modeling part for this analysis.
**Table S1:** The Ameriflux site ID, digital object identifier (DOI), geographic coordinates, elevation (m), International Geosphere‐Biosphere Programme (IGBP) vegetation type, and Köppen Climate class for the NEON eddy covariance towers used in these analyses are presented.
**Table S2:** Description of variables used in the KGML framework.

## Data Availability

All data used in this study are fully open access. Eddy covariance data from NEON sites, along with the cea and fvs partitioned ET components developed by Zahn et al. (2024), are publicly available at https://doi.org/10.5281/zenodo.12191876 (Zahn and Bou‐Zeid 2024a, 2024b). The supporting code and data for the KGML model for reading, processing, and exporting the partitioned datasets is available at https://doi.org/10.5281/ZENODO.18939902 (Ranjbar et al. 2026). All sites used in this study are listed in Table S1, which provides AmeriFlux site IDs, digital object identifiers (DOIs), geographic coordinates, elevation, International Geosphere‐Biosphere Programme (IGBP) vegetation types, and Köppen climate classes for the eddy covariance towers.
