# Supplementary material for: Constrained Carbon Partitioning: A Self‐Trained Physics‐Informed Machine Learning Model Refines GPP Estimates From Eddy Covariance Measurements
Source: Glob Chang Biol. 2026 May 11;32:e70886. doi: 10.1111/gcb.70886 (PMC13161833; doi:10.1111/gcb.70886)
Supplement: Supplementary file 1 — Figure S1: KGML RECO dynamics and method performance in different Plant functional types (PFT). (a) Mean diurnal RECO cycles by IGBP PFT. (b) Seasonal RECO trajectories by day of year using a 14‐day rolling mean. (c) Annual RECO totals (gC m−2 yr.−1; mean ± SD) by PFT across site‐years; tropical/subtropical PFTs are excluded due to limited NEON coverage. (d) Distributions of RECO differences between KGML and nighttime (NT) and daytime (DT) partitioning methods by PFT, shown as boxplots truncated at the 1st–99th percentiles; the dashed line denotes zero difference. CEA partitioning data are used for this analysis. The cea estimates are used in the modeling part for this analysis. Figure S2: Physical soundness and mechanistic consistency of KGML carbon flux estimates. (a) NEE from eddy covariance versus KGML predictions (hexbin, log scale; 1:1 dashed line; R 2, RMSE, bias shown). (b) KGML GPP versus canopy transpiration (binned mean ± SD; linear fit). (c) Stomatal conductance (gs) versus PAR. (d) gs versus VPD (hexbin). (e) WUE (GPP/Transpiration, mmolCO2/H2O) versus VPD. (f) WUE distribution by IGBP biome (boxplots: median, IQR, 1.5× IQR). (g) WUE versus soil moisture. (h) Diurnal WUE (5th–95th percentiles). Data are half‐hourly across NEON sites. The fvs estimates are used in the modeling part for this analysis. Figure S3: Comparative evaluation of KGML against neural network and decision tree partitioning methods. Top row (a–d): GPP comparisons. (a) KGML versus nighttime (NT) neural network. (b) KGML versus daytime (DT) decision tree. (c) NT versus DT. Hexbin plots (log scale) with 1:1 dashed line, linear fit (red), and R 2, RMSE, bias. (d) Mean diurnal GPP for all methods with KGML 5th–95th percentile envelope. Bottom row (e–h): Same layout for RECO. Markers: circles (KGML), squares (NT), triangles (DT). The fvs estimates are used in the modeling part for this analysis. Figure S4: The distributions and differences in GPP estimates across methods. (a) Annual GPP to [file GCB-32-e70886-s001.docx]

**Supplementary Information**

Constrained carbon partitioning: a self-trained physics-informed machine learning model refines GPP estimates from eddy covariance measurements

**Sadegh Ranjbar^1^, Ankur R. Desai^2^, Sophie Hoffman^1^, Einara Zahn^3, 4^, Elie Bou-Zeid^3^, Paul C. Stoy^1^**

^1^Department of Biological Systems Engineering, University of Wisconsin – Madison, Madison, WI, USA.

^2^Department of Atmospheric and Oceanic Sciences, University of Wisconsin – Madison, Madison, WI, USA.

^3^Department of Civil and Environmental Engineering, Princeton University, Princeton, New Jersey, USA.

^4^Department of Earth and Environmental Science, University of Pennsylvania, Philadelphia, PA, USA

Running Title: Physics-informed ML refines GPP estimates

Keywords: Gross primary productivity; Eddy covariance; Knowledge-guided machine learning; Carbon partitioning; Ecosystem respiration; Stomatal physiology; Kok effect; NEON

Corresponding author: Paul Stoy, pcstoy@wisc.edu, Sadegh Ranjbar (sranjbar@wisc.edu)

**Abstract (Supplementary Information)**

This supplementary document provides additional analyses and figures supporting the manuscript “Constrained carbon partitioning: a self-trained physics-informed machine learning model refines GPP estimates from eddy covariance measurements”. It presents detailed evaluations of Gross Primary Production (GPP) and ecosystem respiration (RECO) across plant functional types (PFTs), derived from the Knowledge-Guided Machine Learning (KGML) model, conventional nighttime partitioning (NT), daytime partitioning (DT) methods. Using two independent datasets from conditional eddy accumulation (cea) and flux variance similarity (fvs) with a focus on RECO and fvs results as the main body of the text focuses on GPP and cea. The materials include comparative analyses of daily and annual carbon dioxide fluxes, assessments of model uncertainty, and SHapley Additive exPlanations (SHAP)-based feature importance for GPP and RECO. Contributions of individual physical constraints to model optimization are quantified via gradient norm analysis.

# Text S1:

The diurnal patterns of RECO show distinct temporal dynamics across PFTs (Figure S1a). DBF ecosystems exhibit the most pronounced diurnal variation, with values declining from approximately 3.5 μmol m^-2^ s^-1^ during early morning hours to approximately 2.8 μmol m^-2^ s^-1^ around midday, then rising to approximately 3.2 μmol m^-2^ s^-1^ during afternoon before rising again to approximately 3.5-4.0 μmol m^-2^ s^-1^ in the evening. ENF shows elevated nighttime and early morning RECO (approximately 3.1-3.5 μmol m^-2^ s^-1^) that remains relatively stable with a slight midday dip before recovering in the evening. MF, SAV, and GRA display moderate diurnal patterns, with values ranging between 2.0-2.5 μmol m^-2^ s^-1^, showing afternoon minima around 1.6-1.8 μmol m^-2^ s^-1^. CRO, OSH and WET show the lowest RECO values, with croplands ranging 1.2-1.4 μmol m^-2^ s^-1^ and WET and OSH remaining near 1.0 μmol m^-2^ s^-1^ throughout the day. The seasonal trajectories (Figure S1b) reveal that DBF exhibits the strongest seasonal variation, with RECO rising from approximately 1.0 μmol m^-2^ s^-1^ around day of year 50 to peak values of approximately 6.2 μmol m^-2^ s^-1^ between days 180-210, followed by a sharp decline after day 240. RNF, GRA, and MF also display high seasonal amplitudes with summer peaks around 3-4.5 μmol m^-2^ s^-1^. SAV, OSH and CRO show lower seasonal variation with peak values around 2-3 μmol m^-2^ s^-1^. WET showed the lowest seasonal variation.

Relative annual RECO changes vary considerably across PFTs when comparing KGML against conventional partitioning methods (Figure S1c). For the nighttime partitioning approach (KGML - NT, shown in teal), all PFTs exhibit negative relative changes except WET, indicating that KGML consistently produces lower annual RECO estimates than NT. The largest negative deviations occur in SAV and ENF, with relative changes around -16% to -17%. DBF also shows large negative changes of approximately -15%, while GRA, MF, and OSH exhibit changes around -9% to -10%. CRO shows the smallest negative deviations at approximately -6%. For the daytime partitioning approach (KGML - DT, shown in coral), the pattern also shows predominantly negative relative changes, though generally smaller in magnitude than for NT. ENF shows the largest negative change at approximately -13%, followed by MF and WET at approximately -9% to -7.5%. OSH shows a notable positive relative change of approximately +2%, while CRO, DBF, GRA and SAV show negative changes ranging from approximately -0.5% to -3%.

The distribution of RECO differences between KGML and conventional partitioning methods (Figure S1d) shows that median differences are generally close to zero across most PFTs, with the dashed line indicating zero difference. However, the spread varies considerably by PFT. For KGML minus NT (teal boxes), most PFTs show median differences within ±0.5 μmol m^-2^ s^-1^, though the interquartile range extends to approximately ±2 μmol m^-2^ s^-1^ for several PFTs. For KGML minus DT (coral boxes), the distributions show similar patterns. The full distribution range (1st-99th percentiles) extends to approximately ±2 to ±8 μmol m^-2^ s^-1^ for the most variable PFTs.

# Text S2:

Comparison of RECO estimates between KGML implementations using the cea and fvs approaches (Figure 5 and Figure S3) shows noticeable differences in the magnitude and temporal dynamics of respiration. These differences are substantial, particularly in nighttime periods. However, these differences are largely expected because the fvs formulation relies on photosynthesis-driven constraints, and photosynthesis approaches zero at night in the absence of CAM vegetation as in the case of the ecosystems studied here. Under these conditions, water-use-efficiency–based optimization becomes weakly constrained, which can introduce additional uncertainty in the derived flux partitioning. As a result, there are more missing values in nighttime data in training, testing, and aggregation for visualization in fvs approach. For these reasons, nighttime RECO estimates in the fvs configuration may deviate from those obtained using the cea approach. Importantly, conventional partitioning methods (NT and DT) are also affected by the missing values. To ensure consistency, NaN filtering was applied uniformly across all methods whenever any method contained a missing value; therefore, the comparisons in Figure S3 therefore remain informative for evaluating the relative behavior of the approaches. Addressing these nighttime limitations more explicitly would require introducing additional assumptions and uncertainties into the model.

#
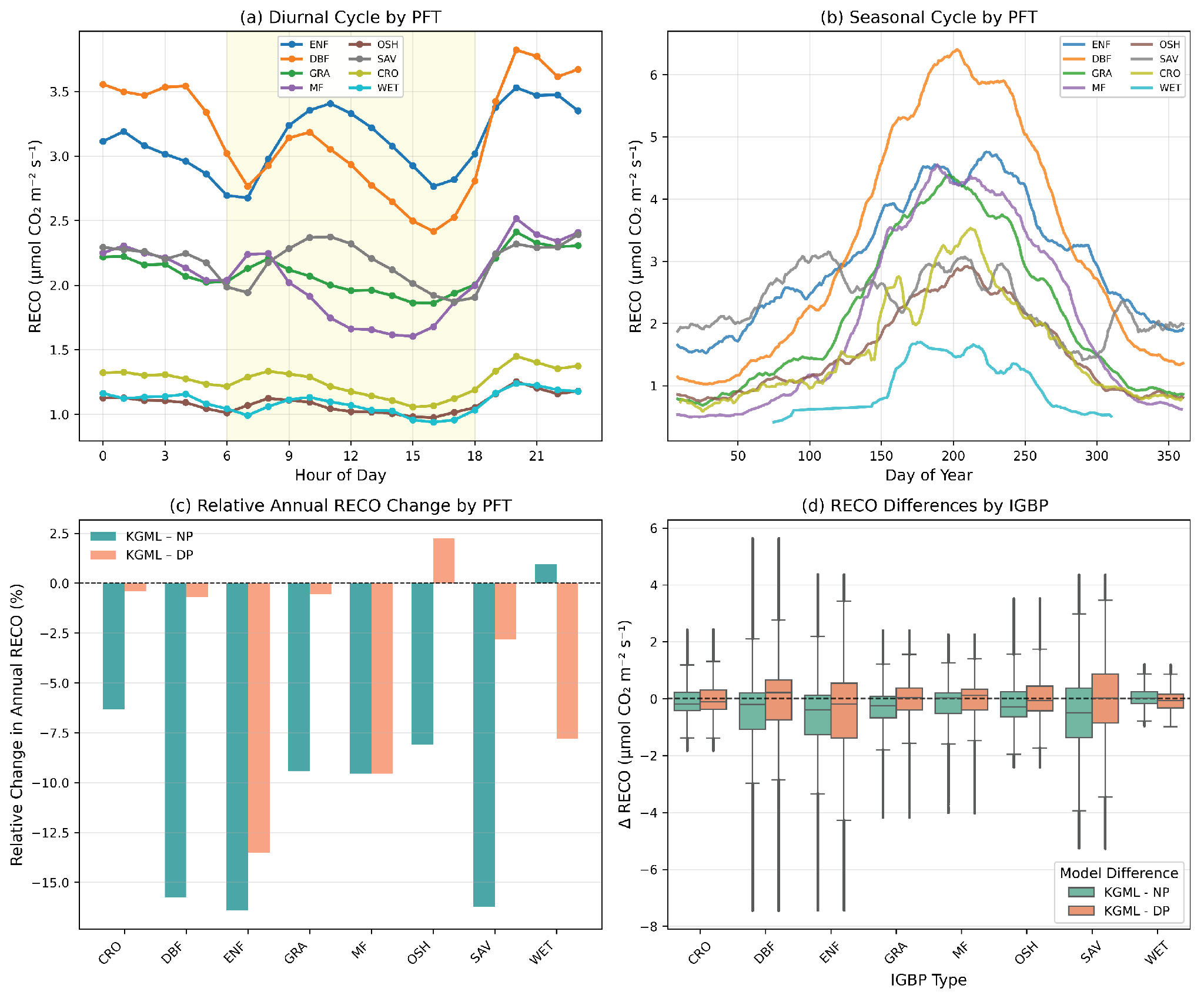


# Figure S1: KGML RECO dynamics and method performance in different Plant functional types (PFT). (a) Mean diurnal RECO cycles by IGBP PFT. (b) Seasonal RECO trajectories by day of year using a 14-day rolling mean. (c) Annual RECO totals (gC m^-2^ yr^-1^; mean ± SD) by PFT across site-years; tropical/subtropical PFTs are excluded due to limited NEON coverage. (d) Distributions of RECO differences between KGML and nighttime (NT) and daytime (DT) partitioning methods by PFT, shown as boxplots truncated at the 1st–99th percentiles; the dashed line denotes zero difference. CEA partitioning data are used for this analysis. The cea estimates are used in the modeling part for this analysis.

#
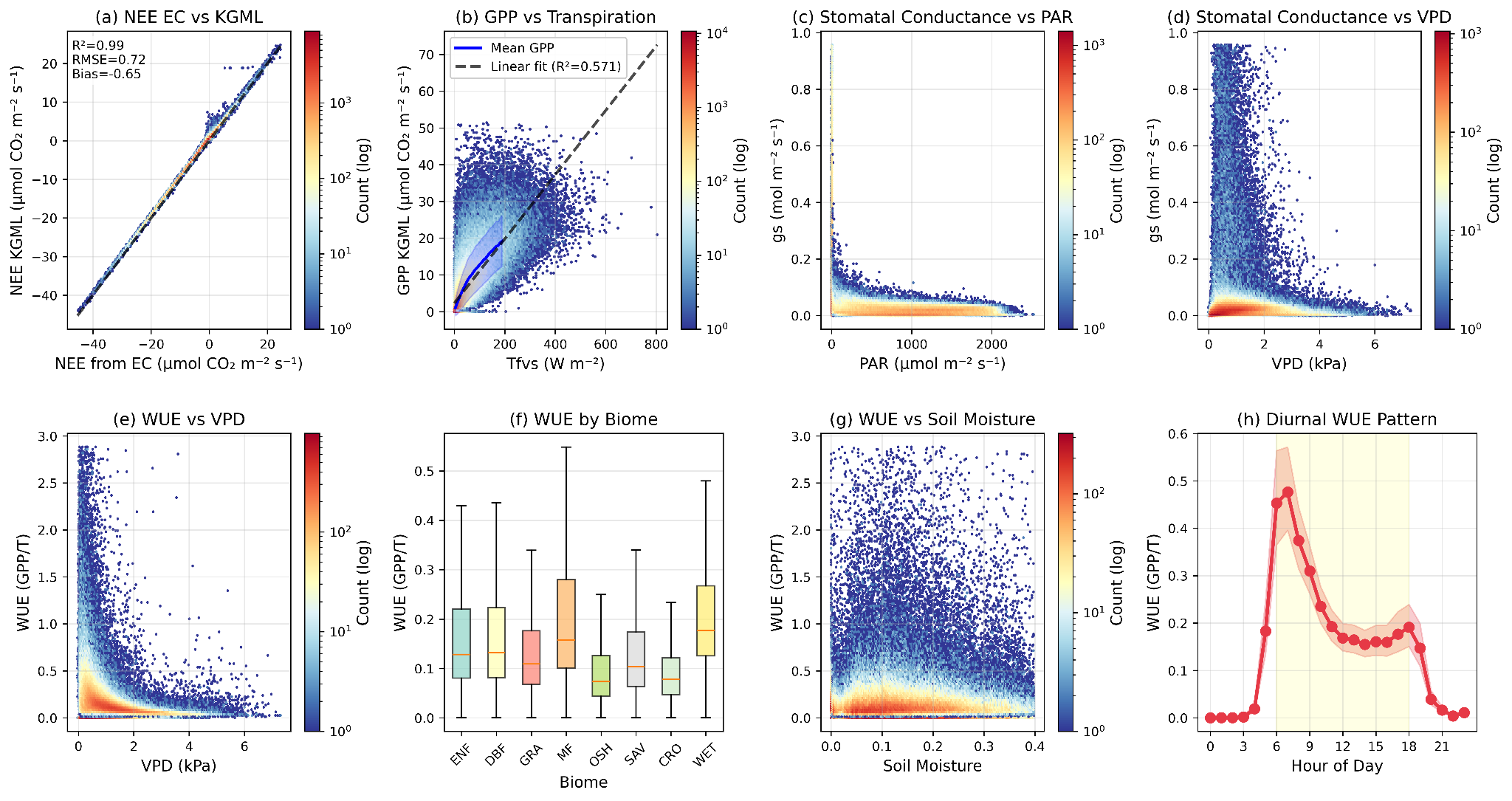
Figure S2: Physical soundness and mechanistic consistency of KGML carbon flux estimates. (a) NEE from eddy covariance versus KGML predictions (hexbin, log scale; 1:1 dashed line; R², RMSE, bias shown). (b) KGML GPP versus canopy transpiration (binned mean ± SD; linear fit). (c) Stomatal conductance (gs) versus PAR. (d) gs versus VPD (hexbin). (e) WUE (GPP/Transpiration, mmolCO_2_​/H_2_​O) versus VPD. (f) WUE distribution by IGBP biome (boxplots: median, IQR, 1.5× IQR). (g) WUE versus soil moisture. (h) Diurnal WUE (5th–95th percentiles). Data are half-hourly across NEON sites. The fvs estimates are used in the modeling part for this analysis.


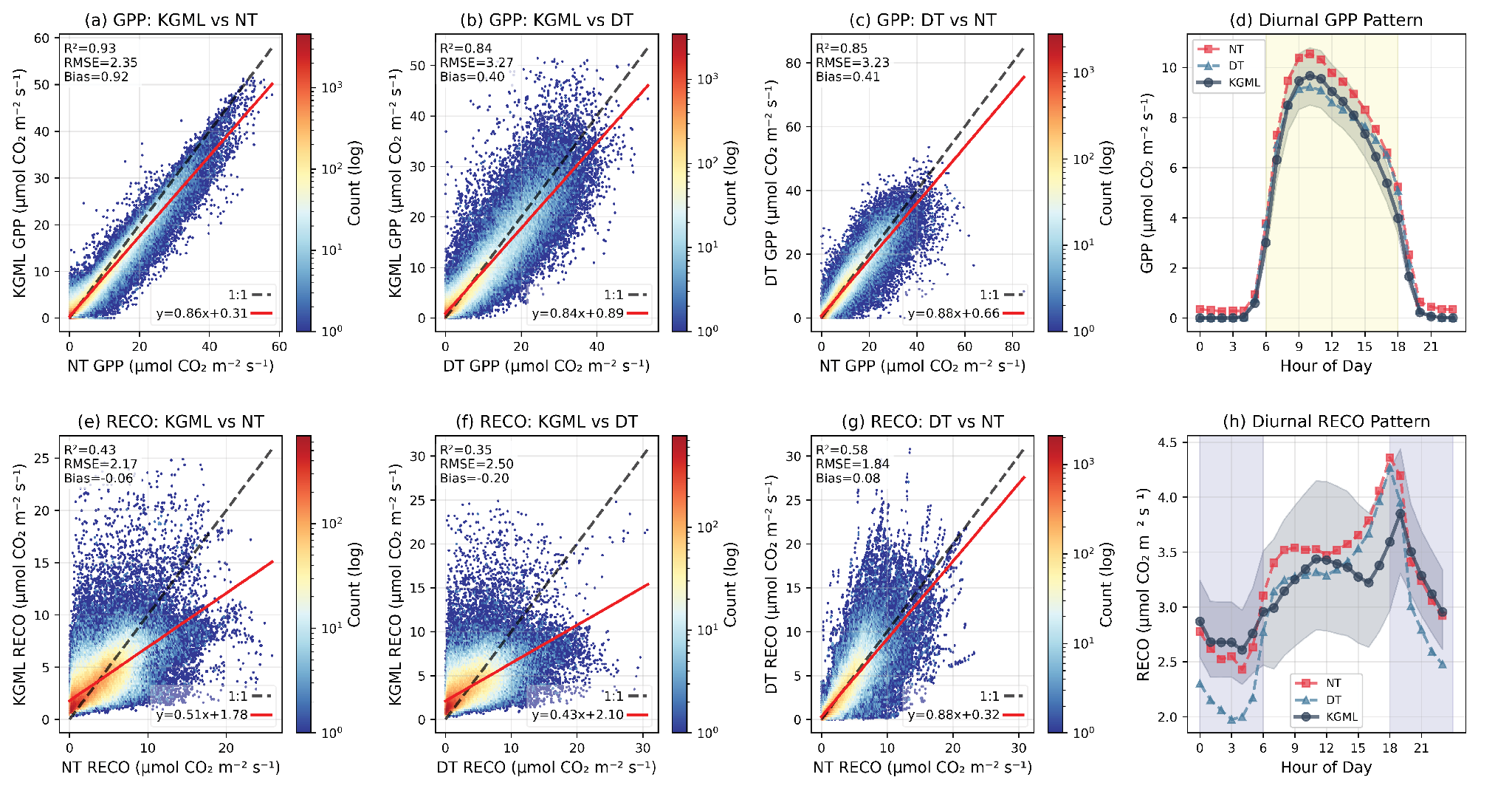


# Figure S3: Comparative evaluation of KGML against neural network and decision tree partitioning methods. Top row (a–d): GPP comparisons. (a) KGML vs. nighttime (NT) neural network. (b) KGML vs. daytime (DT) decision tree. (c) NT vs. DT. Hexbin plots (log scale) with 1:1 dashed line, linear fit (red), and R², RMSE, bias. (d) Mean diurnal GPP for all methods with KGML 5th–95th percentile envelope. Bottom row (e–h): Same layout for RECO. Markers: circles (KGML), squares (NT), triangles (DT). The fvs estimates are used in the modeling part for this analysis.


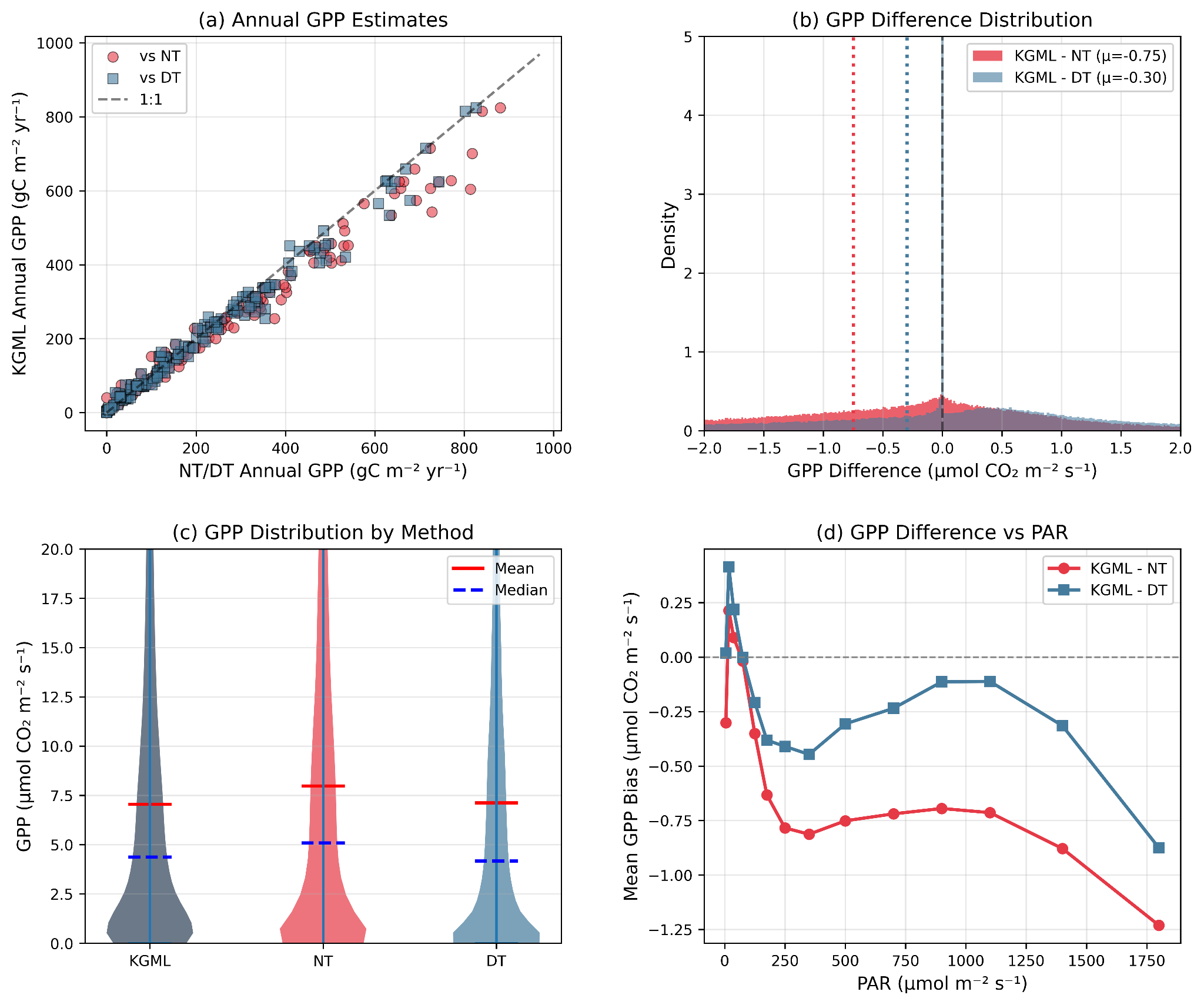


# Figure S4. The distributions and differences in GPP estimates across methods. (a) Annual GPP totals comparing KGML against NT (red circles) and DT (blue squares) methods across site-years. The black dashed line shows 1:1 relationship. (b) Probability density distributions of half-hourly GPP differences (KGML minus NT in red; KGML minus DT in blue). Vertical dashed lines indicate mean bias; the solid black line shows zero difference. (c) Violin plots comparing GPP distributions across methods. (d) Mean GPP bias (from NT/DT) as a function of light availability (PAR bins). Gray dashed line indicates zero bias reference. The fvs estimates are used in the modeling part for this analysis.


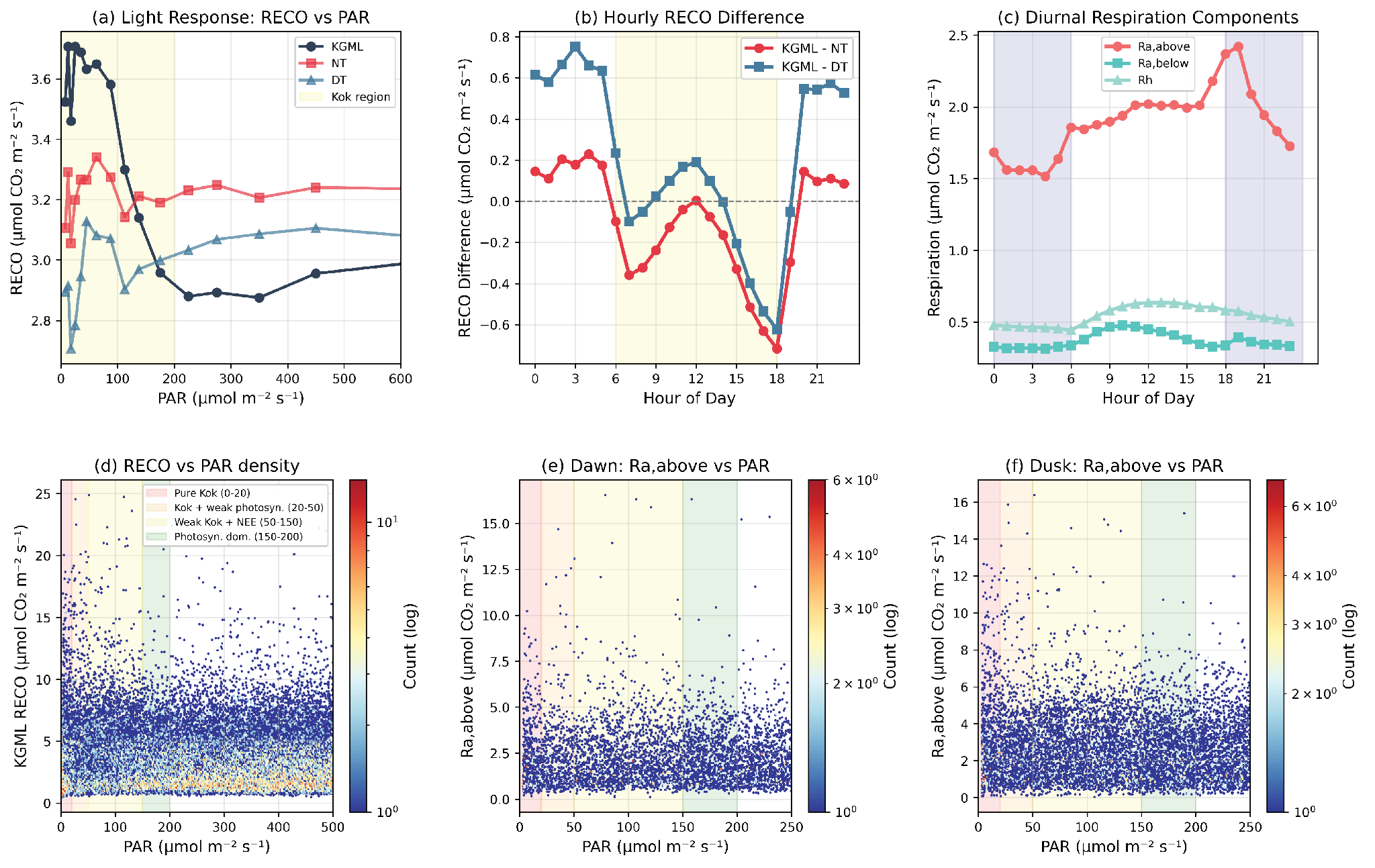


# Figure S5. Mechanistic basis for method differences: Kok effect and light-dependent respiration. (a) RECO versus PAR (<600 μmol m^-2^ s^-1^); yellow shading marks the Kok-sensitive region. (b) Diurnal RECO differences (KGML − NT/DT). (c) KGML respiration components: above-ground autotrophic (Ra,above), below-ground autotrophic (Ra,below), and heterotrophic (Rh). (d) RECO versus PAR (0–500 μmol m^-2^ s^-1^) with PAR regimes color-coded. (e) Dawn relationship between Ra,above and PAR. (f) Dusk relationship between Ra,above and PAR. The fvs estimates are used in the modeling part for this analysis.


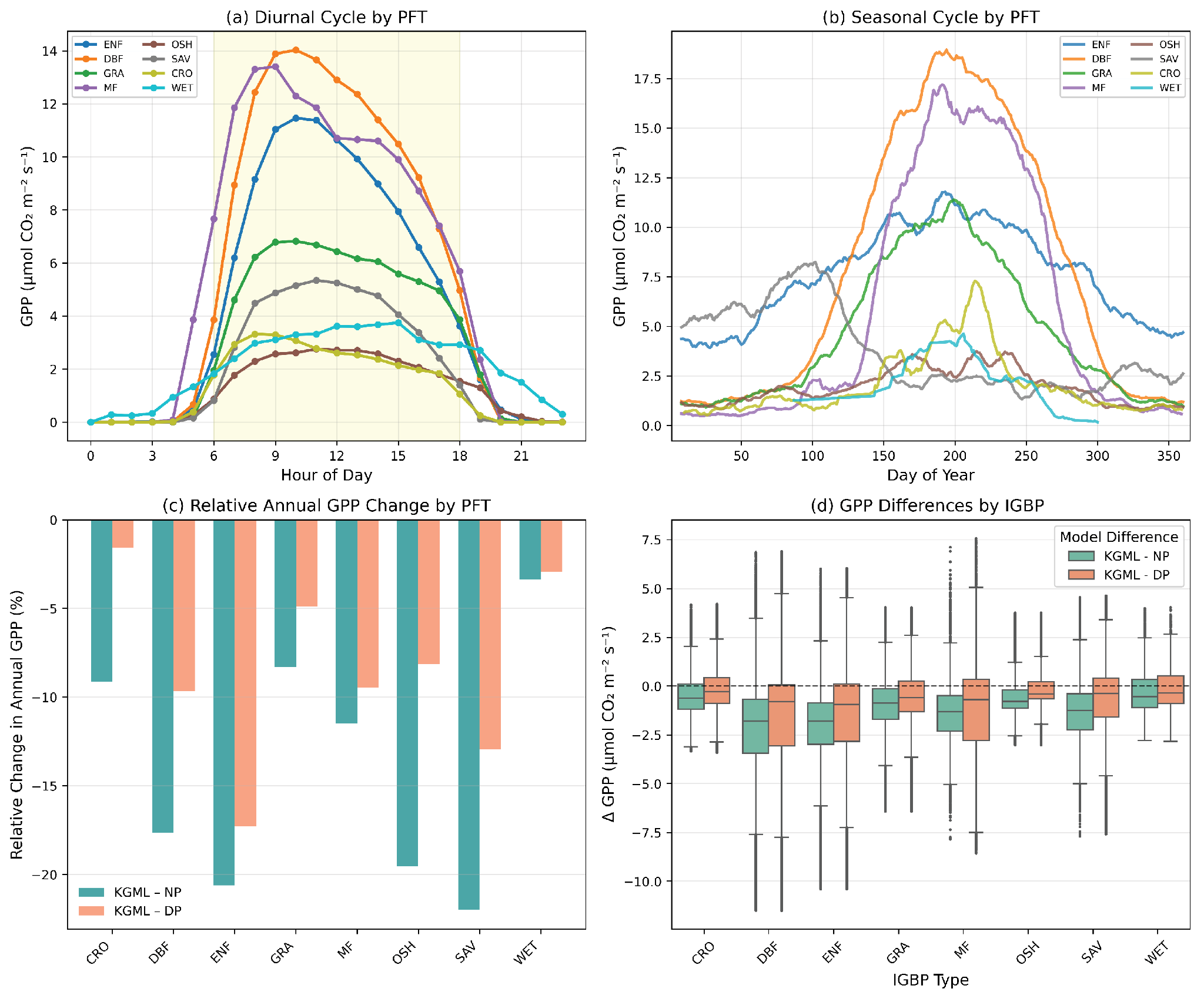


# Figure S6: KGML GPP dynamics and method performance across different plant functional types (PFTs). (a) Mean diurnal GPP cycles by IGBP PFT. (b) Seasonal GPP trajectories by day of year using a 14-day rolling mean. (c) relative changes in annual GPP by PFT. (d) Distributions of GPP differences between KGML and nighttime (NT) and daytime (DT) partitioning methods by PFT, shown as boxplots truncated at the 1st–99th percentiles; dashed line denotes zero difference. The fvs estimates are used in the modeling part for this analysis.


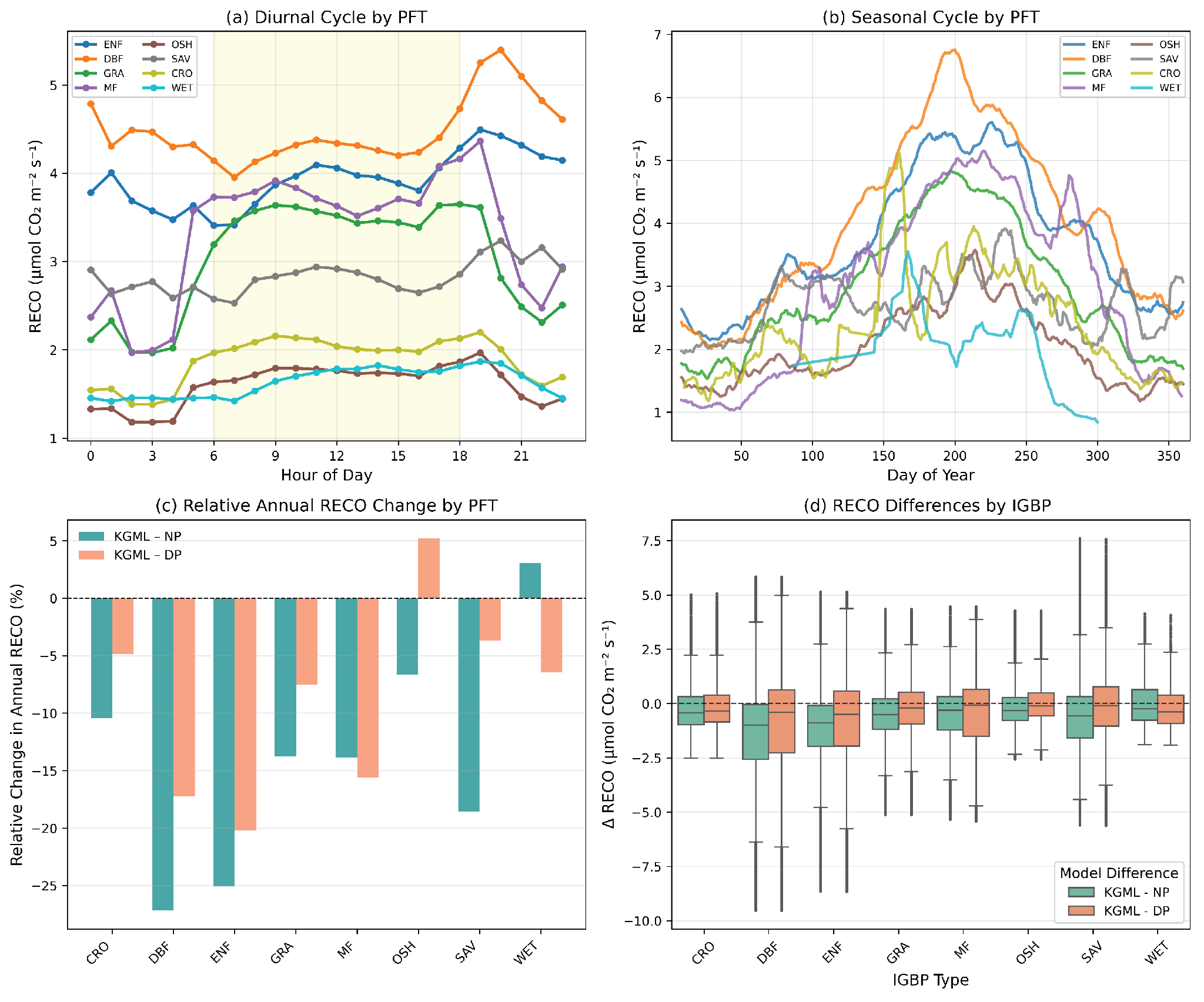


# Figure S7: KGML RECO dynamics and method performance in different Plant functional types (PFT). (a) Mean diurnal RECO cycles by IGBP PFT. (b) Seasonal RECO trajectories by day of year using a 14-day rolling mean. (c) Annual RECO totals (gC m^-2^ yr^-1^; mean ± SD) by PFT across site-years; tropical/subtropical PFTs excluded due to limited NEON coverage. (d) Distributions of RECO differences between KGML and nighttime (NT) and daytime (DT) partitioning methods by PFT, shown as boxplots truncated at the 1st–99th percentiles; dashed line denotes zero difference. CEA partitioning data are used for this analysis. The fvs estimates are used in the modeling part for this analysis.


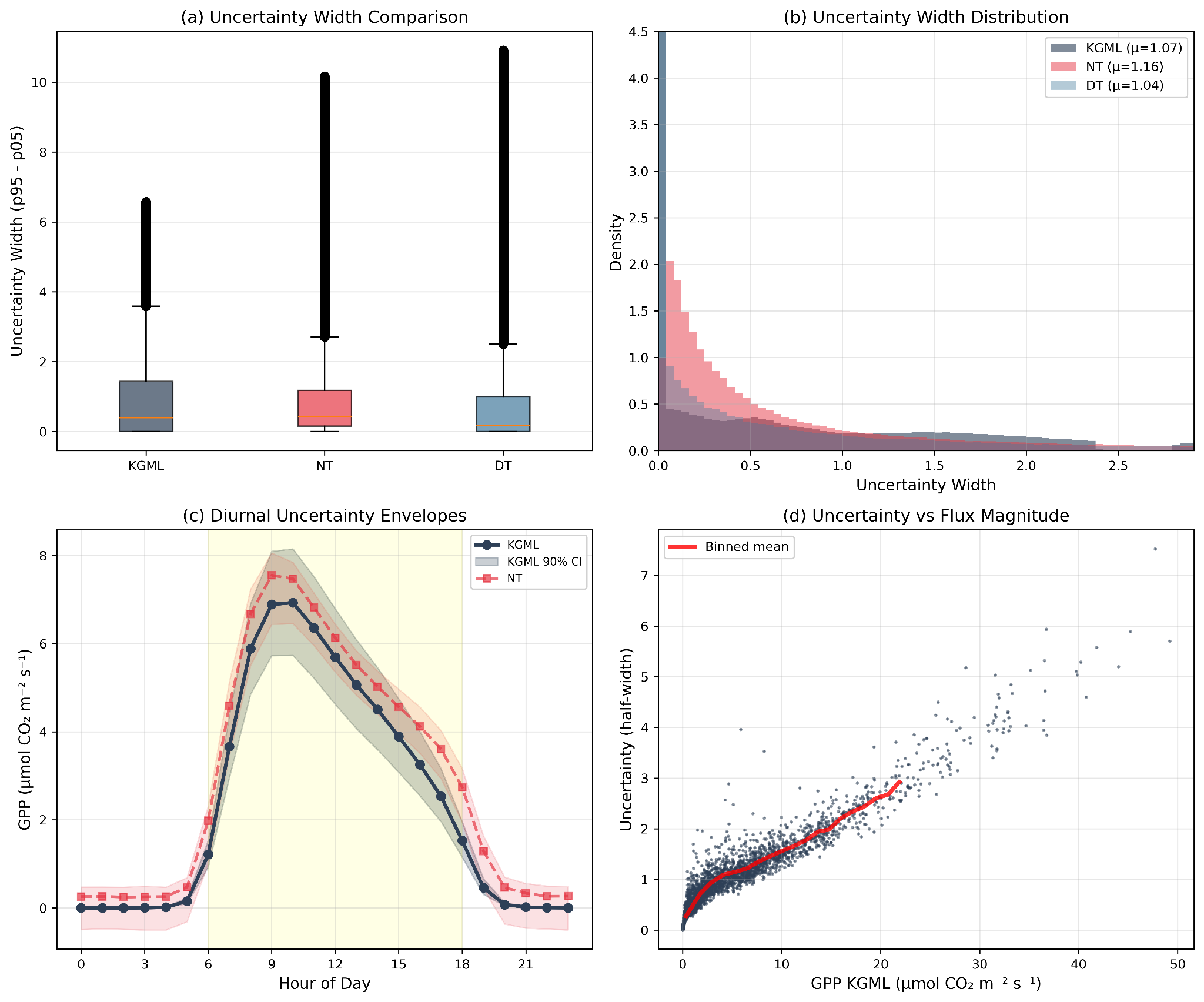


# Figure S8 . Uncertainty quantification and propagation in KGML vs. empirical methods. (a) Distribution of uncertainty width (95th–5th percentile) across methods; KGML from Bayesian ensemble and NT/DT from bootstrap resampling. Boxplots show median, IQR, and 1.5 × IQR whiskers. (b) Probability density of uncertainty widths with vertical dashed lines indicating means. (c) Diurnal GPP mean ± 90% confidence intervals for KGML and NT. (d) KGML uncertainty (half-width of 90% CI) versus flux magnitude with binned mean shown in red. The cea estimates are used in the modeling part for this analysis.


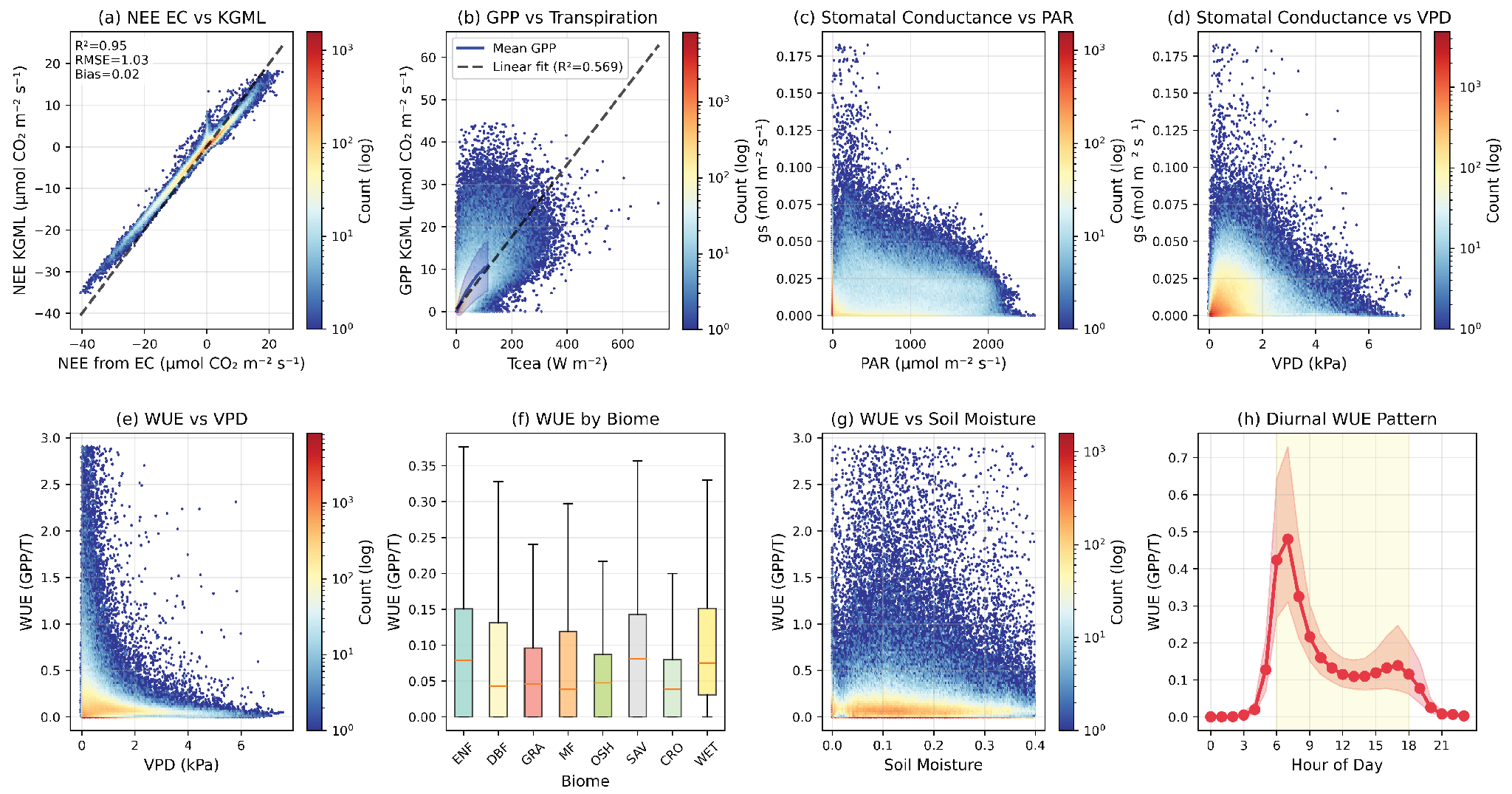


# Figure S9: KGML carbon flux estimates for hold-out test samples. (a) NEE from eddy covariance versus KGML predictions (hexbin, log scale; 1:1 dashed line; R², RMSE, bias shown). (b) KGML GPP versus canopy transpiration (binned mean ± SD; linear fit). (c) Stomatal conductance (gs) versus PAR. (d) gs versus VPD (hexbin). (e) WUE (GPP/Transpiration, mmolCO_2_​/H_2_​O) versus VPD. (f) WUE distribution by IGBP biome (boxplots: median, IQR, 1.5× IQR). (g) WUE versus soil moisture. (h) Diurnal WUE (5th–95th percentiles). Data are half-hourly across NEON sites. The cea estimates are used in the modeling part for this analysis.

# Table S1: The Ameriflux site ID, digital object identifier (DOI), geographic coordinates, elevation (m), International Geosphere-Biosphere Programme (IGBP) vegetation type, and Köppen Climate class for the NEON eddy covariance towers used in these analyses are presented.

| Site ID | DOI | Latitude | Longitude | Elevation (m) | IGBP | Climate |
| --- | --- | --- | --- | --- | --- | --- |
| US-xAB | 10.17190/AMF/1617726 | 45.76 | -122.33 | 363 | ENF | Csb |
| US-xAE | 10.17190/AMF/1671891 | 35.41 | -99.06 | 516 | GRA | Cfa |
| US-xBL | 10.17190/AMF/1671893 | 39.06 | -78.07 | 183 | DBF | Cfa |
| US-xBR | 10.17190/AMF/1579542 | 44.06 | -71.29 | 232 | DBF | Dfb |
| US-xCL | 10.17190/AMF/1671894 | 33.4 | -97.57 | 259 | GRA | Cfa |
| US-xCP | 10.17190/AMF/1579720 | 40.82 | -104.75 | 1654 | GRA | Bsk |
| US-xDC | 10.17190/AMF/1617728 | 47.16 | -99.11 | 559 | GRA | Dfb |
| US-xDL | 10.17190/AMF/1579721 | 32.54 | -87.8 | 22 | MF | Cfa |
| US-xDS | 10.17190/AMF/1671895 | 28.13 | -81.44 | 15 | CVM | Cfa |
| US-xGR | 10.17190/AMF/1634885 | 35.69 | -83.5 | 579 | DBF | Cfa |
| US-xHA | 10.17190/AMF/1562391 | 42.54 | -72.17 | 351 | DBF | Dfb |
| US-xJE | 10.17190/AMF/1617730 | 31.19 | -84.47 | 44 | ENF | Cfa |
| US-xJR | 10.17190/AMF/1617731 | 32.59 | -106.84 | 1329 | OSH | Bsk |
| US-xKA | 10.17190/AMF/1579722 | 39.11 | -96.61 | 1329 | GRA | Cfa |
| US-xKZ | 10.17190/AMF/1562392 | 39.1 | -96.56 | 381 | GRA | Cfa |
| US-xLE | 10.17190/AMF/1773398 | 31.85 | -88.16 | 20 | DBF | Cfa |
| US-xMB | 10.17190/AMF/1671896 | 38.25 | -109.39 | 1767 | OSH | Bsk |
| US-xML | 10.17190/AMF/1671897 | 37.38 | -80.52 | 1126 | DBF | Dfb |
| US-xNG | 10.17190/AMF/1617732 | 46.77 | -100.92 | 578 | GRA | Dfb |
| US-xNQ | 10.17190/AMF/1617733 | 40.18 | -112.45 | 1685 | OSH | Dfb |
| US-xNW | 10.17190/AMF/1671898 | 40.05 | -105.58 | 3513 | ENF | Dfc |
| US-xRM | 10.17190/AMF/1579723 | 40.28 | -105.55 | 2743 | ENF | Dfc |
| US-xRN | 10.17190/AMF/1773400 | 35.96 | -84.28 | 334 | DBF | Cfa |
| US-xSB | 10.17190/AMF/1671899 | 29.69 | -81.99 | 45 | ENF | Cfa |
| US-xSC | 10.17190/AMF/1671900 | 38.89 | -78.14 | 361 | DBF | Cfa |
| US-xSE | 10.17190/AMF/1617734 | 38.89 | -76.56 | 15 | DBF | Cfa |
| US-xSJ | 10.17190/AMF/1671901 | 37.11 | -119.73 | 368 | SAV | Csa |
| US-xSL | 10.17190/AMF/1617735 | 40.46 | -103.03 | 1364 | CRO | Bsk |
| US-xSP | 10.17190/AMF/1617736 | 37.03 | -119.26 | 1160 | ENF | Csa |
| US-xSR | 10.17190/AMF/1579543 | 31.91 | -110.84 | 983 | OSH | Bsk |
| US-xST | 10.17190/AMF/1617737 | 45.51 | -89.59 | 481 | DBF | Dfb |
| US-xTA | 10.17190/AMF/1671902 | 32.95 | -87.39 | 135 | ENF | Cfa |
| US-xTE | 10.17190/AMF/1617738 | 37.01 | -119.01 | 2147 | ENF | Csa |
| US-xTR | 10.17190/AMF/1634886 | 45.49 | -89.59 | 472 | DBF | Dfb |
| US-xUK | 10.17190/AMF/1617740 | 39.04 | -95.19 | 335 | DBF | Cfa |
| US-xUN | 10.17190/AMF/1617741 | 46.23 | -89.54 | 518 | MF | Dfb |
| US-xWD | 10.17190/AMF/1579724 | 47.13 | -99.24 | 579 | GRA | Dfb |
| US-xWR | 10.17190/AMF/1617742 | 45.82 | -121.95 | 407 | ENF | Csb |
| US-xYE | 10.17190/AMF/1617743 | 44.95 | -110.54 | 2116 | ENF | Dfc |

# Table S2. Description of variables used in the KGML framework

| **Variable** | **Units** | **Description** |
| --- | --- | --- |
| H | W m^-2^ | Sensible heat flux from the surface to the atmosphere. |
| USTAR | m s^-1^ | Friction velocity derived from eddy covariance measurements |
| SW_IN | W m^-2^ | Incoming shortwave radiation at the surface. |
| SW_OUT | W m^-2^ | Reflected shortwave radiation from the surface. |
| LW_IN | W m^-2^ | Incoming longwave radiation from the atmosphere. |
| LW_OUT | W m^-2^ | Outgoing longwave radiation emitted by the surface. |
| T_soil1to4 | °C | Averaged soil temperature from multiple soil layers. |
| G_1to5 | W m^-2^ | Soil heat flux averaged across soil heat flux plates. |
| SoilMoisture | m^3^ m^-3^ | Volumetric soil water content measured at NEON sites. |
| AirTemperature | °C | Near-surface air temperature measured at NEON towers. |
| RH | % | Relative humidity measured at NEON towers. |
| PAR | µmol m^-2^ s^-1^ | Photosynthetically active radiation available for photosynthesis. |
| fluxTemp | °C | Sonic temperature associated with flux measurements |
| ustar | m s^-1^ | Friction velocity derived from NEON eddy covariance towers. |
| WindSpeed | m s^-1^ | Horizontal wind speed measured at NEON towers. |
| vpd | kPa | Vapor pressure deficit. |
| NEE | µmol CO_2_ m^-2^ s^-1^ | Net ecosystem exchange of CO_2_ |
| T | mm h^-1^ or kg m^-2^ s^-1^ | Transpiration component of evapotranspiration |
| P | µmol CO_2_ m^-2^ s^-1^ | Photosynthesis component estimated. |
| E | mm h^-1^ or kg m^-2^ s^-1^ | Evaporation component of evapotranspiration. |
| R | µmol CO_2_ m^-2^ s^-1^ | Ecosystem respiration estimated. |
| ET | mm h^-1^ or kg m^-2^ s^-1^ | Total evapotranspiration derived. |
| CO_2_MIXING_RATIO | ppm | Atmospheric CO_2_ concentration measured at the flux tower. |
| IGBP | – | Land cover class based on the IGBP classification system. |
| Mean_NEE | µmol CO_2_ m^-2^ s^-1^ | Mean net ecosystem exchange over the previous three nights |
| NIGHT | Binary (0/1) | Indicator of nighttime conditions (1 = night, 0 = daytime). |
